# Immunological Properties of Murine Parthenogenetic Stem Cells and Their Differentiation Products

**DOI:** 10.3389/fimmu.2017.00924

**Published:** 2017-08-04

**Authors:** Hannah Johannsen, Vijayakumar Muppala, Carina Gröschel, Sebastian Monecke, Leslie Elsner, Michael Didié, Wolfram-Hubertus Zimmermann, Ralf Dressel

**Affiliations:** ^1^Institute of Cellular and Molecular Immunology, University Medical Center Göttingen, Göttingen, Germany; ^2^Institute of Pharmacology and Toxicology, University Medical Center Göttingen, Göttingen, Germany; ^3^DZHK (German Center for Cardiovascular Research), Partner Site Göttingen, Göttingen, Germany

**Keywords:** pluripotent stem cells, natural killer cells, cytotoxic T lymphocytes, major histocompatibility complex class I molecules, natural killer receptor ligands, stem cell differentiation

## Abstract

The perspective to transplant grafts derived from pluripotent stem cells has gained much attention in recent years. Parthenogenetic stem cells (PSCs) are an alternative pluripotent stem cell type that is attractive as source of grafts for allogeneic transplantations because most PSCs are haploidentical for the major histocompatibility complex (MHC). This reduced immunogenetic complexity of PSCs could tremendously simplify the search for MHC-matched allogeneic stem cells. In this study, we have characterized immunological properties of the MHC haploidentical PSC line A3 (H2^d/d^) and the heterologous PSC line A6 (H2^b/d^). Both PSC lines largely lack MHC class I molecules, which present peptides to cytotoxic T lymphocytes (CTLs) and serve as ligands for inhibitory natural killer (NK) receptors. They express ligands for activating NK receptors, including the NKG2D ligand RAE-1, and the DNAM-1 ligands CD112 and CD155. Consequently, both PSC lines are highly susceptible to killing by IL-2-activated NK cells. *In vitro*-differentiated cells acquire resistance and downregulate ligands for activating NK receptors but fail to upregulate MHC class I molecules. The PSC line A6 and differentiated A6 cells are largely resistant to CTLs derived from T cell receptor transgenic OT-I mice after pulsing of the targets with the appropriate peptide. The high susceptibility to killing by activated NK cells may constitute a general feature of pluripotent stem cells as it has been also found with other pluripotent stem cell types. This activity potentially increases the safety of transplantations, if grafts contain traces of undifferentiated cells that could be tumorigenic in the recipient.

## Introduction

Pluripotent stem cells have gained increasing medical interest due to their potential to give rise to any cell type that is affected by degenerative diseases. Cellular grafts, such as retinal cells or cardiomyocytes, can be generated *in vitro* from stem cells in order to treat diseases such as macular degeneration or heart failure (1, 2). Clinical trials to evaluate this potential have meanwhile been initiated (3, 4) and first encouraging results have been reported (5–7). Moreover, not only cells but even complex tissues such as engineered heart muscle (EHM) can be generated *in vitro* from pluripotent stem cells by well-defined procedures and EHMs have been shown in preclinical animal models to augment heart function upon transplantation (8, 9).

In addition to embryonic stem cells (ESCs) (10–12) and induced pluripotent stem cells (iPSCs) (13), further pluripotent stem cell types have been described that could be used as a potential source of grafts, including the so-called multipotent adult germline stem cells (maGSCs) that were generated from the testis of adult mice just by specific cell culture conditions (14). Parthenogenetic stem cells (PSCs) are another pluripotent stem cell type and they can be generated from pharmacologically activated oocytes (15, 16). In view of new transplantation therapies, the different cell types might have specific advantages and disadvantages. The use of ESCs, e.g., is ethically and legally restricted (17) and limited to allogeneic settings whereas iPSCs appear to have a higher risk to carry mutations that were present in the reprogrammed somatic cells or that are introduced during the reprogramming procedure (18, 19). All grafts derived from the various pluripotent stem cell types have in principle the risk to give rise to teratomas, if undifferentiated cells remain present in the grafts even in trace amounts (20). In immunodeficient mice, transplantation of 2 murine and 245 human ESCs have been reported to be sufficient to result in teratoma growth (21, 22).

Another problem associated with transplantations of pluripotent stem cell-derived cells or tissues is the immune rejection of allogeneic and possibly (due to the expression of developmental or neo-antigens) even autologous grafts (23). Terminally differentiated grafts were tolerated in syngeneic recipients (24–26). However, therapeutically relevant grafts that were obtained by *in vitro* differentiation procedures from human iPSCs have recently been reported to be at risk for rejection by the “autologous” immune system in humanized mice depending on the cell types into which the iPSCs had been differentiated before transplantation (27). Nonetheless, autologous grafts would have substantially higher chances to be tolerated by the recipients without requiring immunosuppression or immunomodulation than allogeneic grafts. Although autologous grafts can be in principle obtained from human iPSCs, strategies for an autologous therapy face challenges imposed by regulatory requirements, high costs, and the long duration of the procedures if starting with the reprogramming of somatic cells to iPSCs that would restrict the therapy to diseases that do not require a swift treatment.

Banking of human leukocyte antigen (HLA)-typed and well-characterized stem cells might be an alternative to generate grafts that are matched at least for major histocompatibility complex (MHC) class I antigens (4, 28, 29), which are expected to be most important for the rejection of stem cell-derived grafts. In this respect, PSCs are a highly interesting pluripotent stem cell type because they are derived from haploid oocytes and are even in a subsequent diploid state typically homozygous for the MHC region. However, depending on the method used for parthenogenetic activation and due to crossing over in meiosis I, genomic regions close to the telomere can be heterozygous (30). Homozygosity in the HLA complex on chromosome 6 would greatly reduce the immunogenetic complexity of PSCs and enable targeting of a large proportion of patients with a limited number of PSC lines at least in some populations (2, 29).

Murine PSCs have been shown to differentiate into cardiomyocytes similarly as other pluripotent stem cell types (31). The PSC line A3 derived from a (C57BL/6J × DBA/2J) F1 (B6D2F1) mouse (H2^b/d^), which carries a homologous MHC region on chromosome 17 (H2^d/d^), has been used previously to generate EHMs and to treat heart failure in a preclinical mouse model. Upon MHC-matched transplantation into DBA/2J mice with a myocardial infarction, these EHMs improved the regional myocardial function and the transplants survived in the recipients receiving only methylprednisolone (5 mg/kg/day) for immunosuppression (31).

In parallel to these experiments, the immunogenicity of the PSC line A3 (H2^d/d^) and the MHC heterologous PSC line A6 (H2^b/d^) has been evaluated in teratoma assays (31). Both lines formed teratomas in immunodeficient SCID mice as well as in immunocompetent syngeneic B6D2F1 mice (H2^b/d^) indicating pluripotency. DBA/2J (H2^d^), Balb/c (H2^d^), and C57BL/6 (H2^b^) recipients regularly rejected PSC A6 cells, for which one MHC haplotype of the PSC A6 cells is mismatched. PSC A3 cells were rejected by MHC-mismatched C57BL/6 recipients. However, they formed teratomas in MHC-matched DBA/2J and Balb/c mice, despite a minor histocompatibility antigen (miHAg) mismatch in Balb/c recipients and a partial miHAg-mismatch in DBA/2J mice. PSC A3 cells also formed teratomas in B6D2F1 chimeras despite lacking the H2^b^ haplotype, which might contribute to the inhibition of natural killer (NK) cells in the B6D2F1 mice (31). These results suggested that PSCs can engraft across barriers of miHAgs and in case of a missing H2 haplotype in the absence of any immunosuppressive therapy. Notably, this finding was at variance to several studies investigating ESCs (20). We found that the ESC line MPI-II, e.g., formed teratomas in syngeneic 129Sv mice (H2^b^) but was rejected by C57BL/6 recipients (H2^b^) that are mismatched for miHAgs only (32) and others reported similar results for other ESC lines (33–35). These findings raised the question whether the immunological properties of PSCs are different from other pluripotent stem cell types and might explain the differences in the outcome of teratoma assays in recipients mismatched for miHags.

So far, the analysis of the immunological properties of PSCs is lacking behind the other pluripotent stem cell types. We have shown previously that mouse ESCs, iPSCs, and maGSCs are targets for IL-2-activated allogeneic as well as syngeneic NK cells (32, 36, 37) and that NK cells can impair the growth of teratomas upon transplantation of the stem cells (20, 36, 38). Since *in vitro* differentiation increased the resistance of these pluripotent stem cell types (36), NK cells might increase the safety of transplantations because they can target undifferentiated cells while ignoring differentiated cells. Moreover, ESCs, iPSCs, and maGSCs were killed by peptide-specific cytotoxic T lymphocytes (CTLs) upon pulsing of the targets with the appropriate peptide despite being negative for MHC class I molecules at least at a level detectable by flow cytometry (39).

We now determined the susceptibility of the two previously described PSC lines, PSC A3 and PSC A6 (31), of which one carries a homologous MHC region (H2^d/d^) and the other is heterologous (H2^b/d^), as well as differentiated cells derived from these cell lines to NK cells and peptide-specific CTLs from T cell receptor (TCR) transgenic OT-I mice. As the other pluripotent stem cell types, the PSCs were susceptible to killing by activated NK cells and differentiation decreased the killing. Thus, susceptibility to killing by NK cells is a feature of all pluripotent stem cell types that have been investigated so far and this feature might have interesting implications for the therapeutic application of grafts derived from pluripotent stem cells.

## Materials and Methods

### Cell Lines and Cell Culture

The PSC lines A3 and A6 have been previously described and characterized as pluripotent stem cells (31). They had been generated from oocytes of (C57BL/6J × DBA/2J) F1 mice (B6D2F1) by activation with strontium chloride and inhibition of the second polar body extrusion with cytochalasin B. The blastocysts were transferred onto mitomycin C-inactivated mouse embryonic fibroblasts (MEFs) and inner cell mass outgrowths were manually selected at day 5 and sequentially further cultured and dispersed to establish cell lines. Since PSCs are largely homozygous, the line A3 carries the H2^d^ haplotype on both chromosomes 17. Line A6, in contrast, is heterozygous for the MHC region due to a crossing over during meiosis I and carries both the H2^d^ and H2^b^ haplotypes (31). Both PSC lines were cultured on inactivated MEFs in Dulbecco’s modified Eagle’s medium (DMEM) (Gibco, Thermo Fisher Scientific, Waltham, MA, USA) with high glucose, no pyruvate, and 25 mmol/l N-2-hydroxyethylpiperazine-N′-2-ethanesulfonic acid. The stem cell medium was supplemented with 20% fetal calf serum (FCS), 1,000 U/ml leukemia inhibitory factor (Merck, Darmstadt, Germany), 2 mmol/l glutamine, 1× non-essential amino acids, 50 U/ml penicillin, 50 μg/ml streptomycin, 1 mmol/l Na-pyruvate, 30 μmol/l adenosine, 30 μmol/l guanosine, 30 μmol/l cytidine, 30 μmol/l uridine, 10 μmol/l thymidine, and 100 μmol/l 2-mercaptoethanol. The PSC colonies were dissociated every 48–72 h in 0.25% trypsin/EDTA and split at a ratio of 1:3 or 1:6. Before being used for experiments, the cell suspensions were preplated for 30 min on tissue culture plates to reduce the content of MEFs. The mouse cell lines YAC-1 (H2^a^), RMA (H2^b^), and X63-Ag8.653 (H2^d^) were cultured as described previously (36, 39).

### Differentiation of PSCs

For *in vitro* differentiation of PSCs, the hanging drop method was applied as described previously (31). Briefly, hanging drops containing 500 cells in 20 μl Iscove’s medium (Gibco) supplemented with 20% FCS, 2 mmol/l glutamine, 1× non-essential amino acids, 50 U/ml penicillin, 50 μg/ml streptomycin, and 100 μmol/l 2-mercaptoethanol were cultured for 2 days. In this time embryoid bodies had formed and were subsequently transferred into Petri dishes for a 5-day suspension culture period before final transfer to cell culture dishes for further 5 days. At day 12, the *in vitro*-differentiated cells were harvested with 0.25% trypsin/EDTA and used for experiments.

### Generation of Cytotoxic Effector Cells

Lymphokine-activated killer (LAK) or NK cells were obtained from spleens of 129Sv, Balb/c, C57BL/6, DAB/2J, and FVB.N mice. CTLs were derived from spleens of TCR-transgenic OT-I mice (40) that recognize the peptide SIINFEKL in a H2K^b^-restricted manner. All mice were bred in the central animal facility of the Medical University Center Göttingen. They were narcotized in CO_2_ and killed by cervical dislocation before spleens were taken aseptically. Lymphocytes were obtained from the spleens using a Tenbroeck homogenizer. Erythrocytes were removed from splenocytes by incubation for 5 min in lysis buffer (155 mM NH_4_Cl, 10 mM KHCO_3_, 0.1 mM EDTA, pH 7.4–7.8). To obtain LAK cells, the splenocytes were then cultured for 4 days in DMEM supplemented with 10% FCS, 50 μM 2-mercaptoethanol, and 1,000 U/ml IL-2 (Immunotools, Friesoythe, Germany). Untouched mouse NK cells were isolated by magnetic-activated cell sorting (MACS) using a mouse NK cell kit (Miltenyi Biotec, Bergisch Gladbach, Germany) for negative selection according to the manufacturer’s instruction. The purity of the isolated CD49b^+^CD3^−^ NK cells was always determined by flow cytometry and found to be >90% in all experiments. For activation, the purified NK cells were then cultured for 4 days similarly as the LAK cells. Splenocytes from naïve OT-I mice were stimulated *in vitro* in the presence of 1 nM SIINFEKL peptide (Ovalbumin 257–264; Bachem Biochemica, Heidelberg, Germany) as previously described (41) before being used as effector cells.

### ^51^Chromium Release Assays

Target cells were labeled by incubating 1 × 10^6^ cells in a suspension of 200 μl DMEM, 100 μl FCS, and 50 μCi Na_2_^51^CrO_4_ (Hartmann Analytic, Braunschweig, Germany) for 1 h at 37°C and then washed three times with DMEM. These ^51^chromium-labeled target cells (5 × 10^3^ per well of round-bottomed microtiter plates) were cocultured with LAK or NK cells for 4 h at 37°C in triplicates at several effector to target (E:T) ratios in 200 μl of DMEM containing 10% FCS. To allow for a peptide-specific killing by CTLs derived from OT-I mice, the respective wells were supplemented with 0.5 μg/ml SIINFEKL peptide and to determine the calcium dependency of killing, 2 mM ethyleneglycol-bis(β-aminoethyl ester)-*N*,*N*,*N*′,*N*′-tetraacetic acid (EGTA) and 4 mM MgCl_2_ were added to additional wells. The microtiter plates were centrifuged for 5 min at 40 × *g* before supernatants and sediments were separately taken to determine radioactivity in each well using a Wallac MicroBeta Trilux counter (PerkinElmer Life Sciences, Rodgau Jügesheim, Germany). Spontaneous release was determined by incubating target cells without the effector cells. The percentage of specific lysis of the target cells was calculated as described previously (42).

### Flow Cytometry

Flow cytometry was performed on a FACS Calibur flow cytometer (BD Biosciences, Heidelberg, Germany) using CellQuestPro data acquisition and analysis software. The antibodies (Abs) used for flow cytometry are described in Table S1 in Supplementary Material. Isotype controls were used for directly labeled monoclonal antibodies (mAbs). For staining, 2 × 10^5^ cells were incubated in 100 μl phosphate-buffered saline (PBS) with 1 μg of the respective primary mAb for 30 min at 4°C before washing with PBS. To detect the unlabeled mAbs, the cells were incubated subsequently in 100 μl PBS with 1 μl of a fluorescein isothiocyanate (FITC)-labeled goat anti-rat IgG Ab (for anti-CD112, anti-CD155, and anti-RAE-1) or FITC-labeled goat anti-mouse IgM Ab (for anti-SSEA-1) for 30 min. The biotinylated anti-Qa-1 mAb was detected with PE/Cy5-labeled streptavidin (Biolegend). In these experiments, cells stained with the secondary reagent only served as control. Recombinant mouse NKG2D-human IgG_1_-Fc (139-NK; R&D Systems, Wiesbaden, Germany), mouse 2B4-human IgG_1_-Fc (3514–2B; R&D Systems), and mouse NKp46-human IgG_1_-Fc (2225-NK; R&D Systems) chimeric proteins were used to detect cell surface expression of NKG2D, 2B4, and NKp46 ligands, respectively. A polyclonal FITC-conjugated goat anti-human IgG served as a secondary reagent. A recombinant mouse DNAM-1-mouse IgG_2a_-Fc chimeric protein (4436-DN; R&D Systems) was used to detect DNAM-1 ligands. A polyclonal FITC-conjugated goat anti-mouse IgG was used as a secondary reagent. The percentage of positive cells expressing the analyzed molecule and their mean fluorescence intensity (MFI) was determined by subtracting the values of the appropriate controls.

### Gene Expression Analysis

Total RNA was extracted from cell lines or tissues using the TRIZOL reagent (Invitrogen, Carlsbad, CA, USA) according to the manufacturer’s instructions. The RNA was then treated with RNase free DNase (RQ1, Promega, Madison, WI, USA) for 20 min at 37°C and purified by phenol–chloroform–isoamyl alcohol (25:24:1) extraction and precipitated with 1/10 volume 300 mM sodium acetate (pH 4.8) and 1 volume 2-propanol before washing with 70% ethanol and solving in RNase free water. The quantity of the extracted RNA was determined with a ND-1000 spectrophotometer (NanoDrop Technologies, Wilmington, DE, USA). For synthesis of cDNA random oligo primers (Promega, Madison, WI, USA) were used. The reverse transcription of RNA was performed for 60 min at 37°C with M-MLV RT polymerase (Promega, Madison, WI, USA) in a total volume of 25 μl. Gene expression levels were analyzed by quantitative real-time polymerase chain reaction assays using the forward and reverse primers given in Table S2 in Supplementary Material. The mRNA expression of the housekeeping gene hypoxanthine guanine phosphoribosyl transferase 1 (*Hprt1*) was always monitored as internal control. Amplification reactions were carried out in 96-well plates in 25 μl reaction volumes with the Power SYBR green PCR master mix (Applied Biosystems, Foster City, CA, USA). The PCR plates were preheated for 2 min at 50°C and for 10 min at 95°C followed by 40 cycles of denaturation (15 s at 95°C), and amplification (1 min at 60°C). All reactions were performed in technical triplicates using an ABI 7500 Real Time PCR System. For the data analysis, the ABI 7500 SDS software (Applied Biosystems) was used. The variations in cDNA concentration in different samples were normalized to the housekeeping gene *Hprt1*. The relative level of transcripts was then indicated as the delta cycle threshold (Δct) value (ct for the gene of interest minus ct for *Hprt1*).

### Statistical Analysis

Results are shown as mean with SD or as mean with SEM for cytotoxicity assays. The data were evaluated with the SPSS software (IBM, Armonk, NY, USA). Excel or the GraphPad Prism (GraphPad Software, La Jolla, CA, USA) programs were used to generate graphs. Analyses of variance (ANOVA) adjusted for repeated measures at different E:T ratios were used to evaluate the cytotoxicity data for differences with respect to target and/or effector cell types and Bonferroni tests were selected as *post hoc* tests. Non-parametric Kruskal–Wallis (*H*) tests were used to compare flow cytometric data sets that included non-normally distributed target variables or variables with few data points. Dunn’s or Wilcoxon (*U*) tests were used as *post hoc* test to compare two sets of data. A *P*-value of ≤0.05 in two-sided tests was considered significant. Significance levels are shown in the following way, if not the exact *P*-value is given: *P ≤ 0.05; **P ≤ 0.01.

## Results

### PSCs Are Targets for Syngeneic and Allogeneic LAK and NK Cells

**Figure 1:**
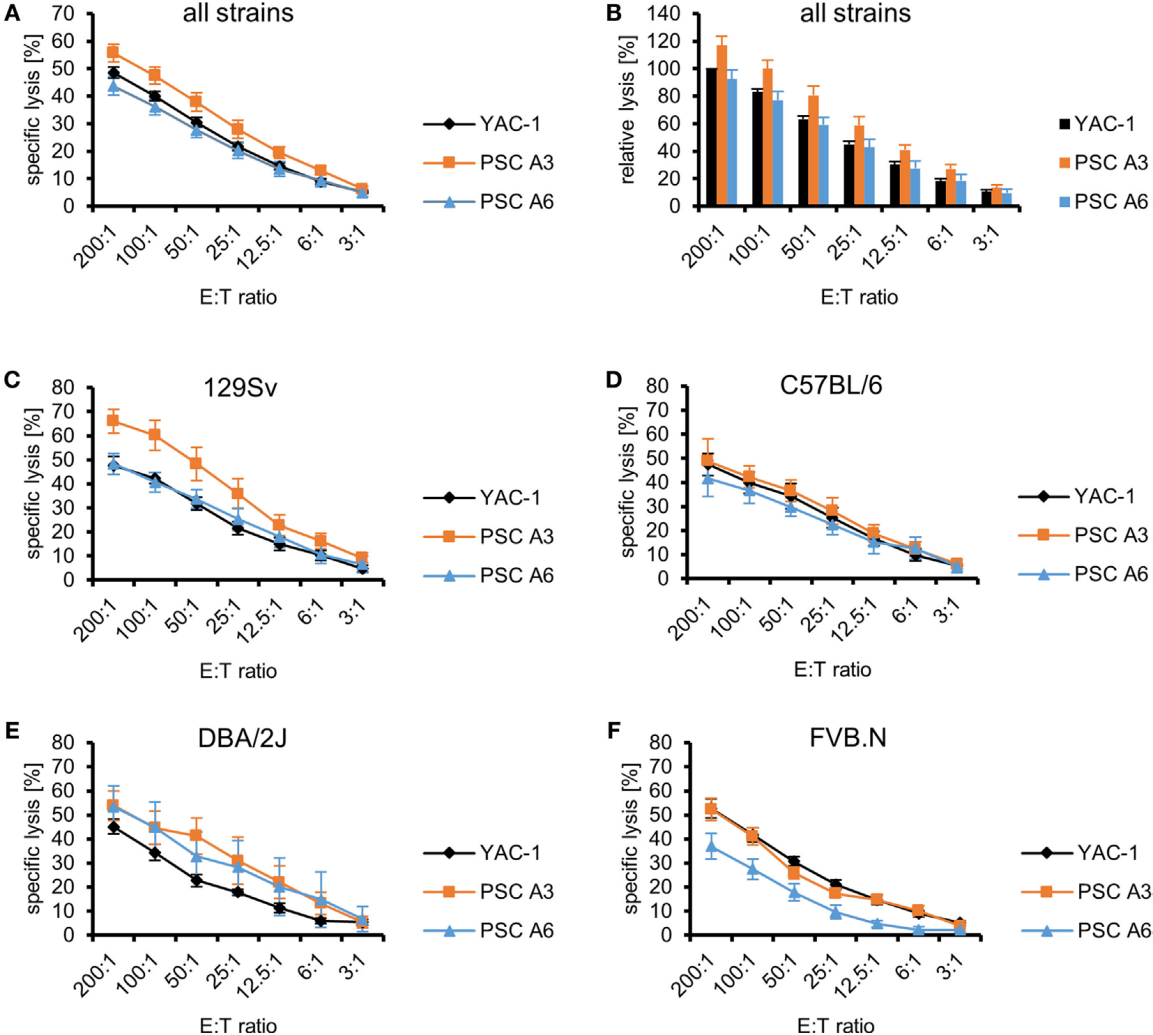
Comparison of the killing of parthenogenetic stem cell (PSC) A3, PSC A6, and YAC-1 target cells by lymphokine-activated killer (LAK) cells of four mouse strains. **(A)** A summary of means of specific lysis and the SEM is shown as determined by ^51^Cr-release assays of the three target cell lines YAC-1 (*n* = 38), PSC A3 (*n* = 18), and PSC A6 (*n* = 20) using LAK cells from four mouse strains (129Sv, *n* = 10; C57BL/6, *n* = 10; DBA/2J, *n* = 7; FVB.N, *n* = 11). **(B)** The data are shown as relative lysis calculated by setting the specific lysis of YAC-1 cells at the highest effector to target (E:T) ratio (200:1) in individual experiments to 100% and adjusting the relative lysis at lower E:T ratios and of other target cells accordingly. **(C)** Summary of means of specific lysis and the SEM by LAK cells derived from 129Sv mice (*n* = 10) of YAC-1 (*n* = 10), PSC A3 (*n* = 5), and PSC A6 cells (*n* = 5). **(D)** Summary of means of specific lysis and the SEM by LAK cells derived from C57BL/6 mice (*n* = 10) of YAC-1 (*n* = 10), PSC A3 (*n* = 4), and PSC A6 cells (*n* = 6). **(E)** Summary of means of specific lysis and the SEM by LAK cells derived from DBA/2J mice (*n* = 7) of YAC-1 (*n* = 7), PSC A3 (*n* = 4), and PSC A6 cells (*n* = 3). **(F)** Summary of means of specific lysis and the SEM by LAK cells derived from FVB.N mice (*n* = 11) of YAC-1 (*n* = 11), PSC A3 (*n* = 5), and PSC A6 cells (*n* = 6).

We have previously shown that several murine pluripotent stem cell types, including ESCs, iPSCs, and maGSCs, are targets for LAK as well as purified IL-2-activated NK cells (36). Therefore, we now determined the susceptibility of PSCs, as a further pluripotent stem cell type, to LAK and NK cells. Two PSC lines A3 (H2^d^) and A6 (H2^b/d^) were investigated and compared to the typical NK target cell line YAC-1 (H2^a^) in ^51^Cr-release assays as shown exemplarily in Figure S1A in Supplementary Material with LAK effector cells from 129Sv mice. Independent experiments were done at different days with effector cells obtained from different mice. To allow for a better comparison with our previously published results on other pluripotent stem cell targets (36), the specific lysis of YAC-1 cells at the highest E:T ratio (200:1) was set to 100% in an additional analysis and the relative lysis at lower E:T ratios and of other target cells was calculated accordingly (Figure S1B in Supplementary Material). In general, all targets (PSC A3, PSC A6 and YAC-1 cells) were killed by LAK cells but their specific lysis varied (*P* < 0.0001, ANOVA). Overall, PSC A3 cells were killed better than PSC A6 cells (*P* < 0.0001) and YAC-1 cells (*P* < 0.0001, Bonferroni *post hoc* test). The specific lysis of PSC A6 cells was in similar range as YAC-1 control cells (Figure 1A). The relative lysis of the PSC targets in relation to YAC-1 cells is shown in Figure 1B to allow for comparison with the killing of other pluripotent stem cell types (36).

Lymphokine-activated killer cells from four mouse strains (129Sv, C57BL/6, DBA/2J, and FVB.N) were used as effector cells in these experiments (Figures 1C–F). These strains have been selected because LAK cells from 129Sv, C57BL/6, and FVB.N mice had been used previously to assess the susceptibility of ESCs, iPSCs, and maGSCs to LAK cell-mediated killing (36) and DBA/2J mice were included in addition because they are the second parental strain of the two PSC lines. The inbred strains represent together some of the genetic variability of mice. We compared the susceptibility of the target cell lines to LAK cells derived from the four strains and indeed found overall significant differences (*P* < 0.0001, ANOVA). The LAK cells differed only slightly in their ability to kill YAC-1 control cells (*P* = 0.012, ANOVA) with DBA/2J being less efficient than C57BL/6 and FVB.N LAK cells (*P* < 0.05, Bonferroni *post hoc* tests) (Figure S2A in Supplementary Material). Differences of the ability of LAK cells of the four mouse strains to kill the PSC lines were more pronounced (PSC A3: *P* < 0.0001, PSC A6: *P* < 0.0001, ANOVA). PSC A3 cells were more susceptible to LAK cells from 129Sv mice than C57BL/6 (*P* = 0.004) and FVB.N mice (*P* < 0.0001, Bonferroni *post hoc* test). PSC A6 cells were more resistant to LAK cells from FVB.N mice than from the other strains (*P* < 0.05, Bonferroni *post hoc* tests). Moreover, the PSC A3 cells were lysed more efficiently than PSC A6 cells by LAK cell from 129Sv (*P* < 0.0001, ANOVA) and FVB.N mice (*P* < 0.0001), whereas the specific lysis of the two PSC lines did not differ with C57BL/6 (*P* = 0.1332) and DBA/2J (*P* = 0.7324) effector cells. The relative lysis of the targets by effector cells of the four strains is shown in Figures S2B–E in Supplementary Material to allow for comparison with previously published data on other pluripotent stem cell types (36). The 129Sv and C57BL/6 mice share the MHC haplotype H2^b^ with A6 cells, DBA/2J mice share the H2^d^ haplotype with A3 and A6 cells, and FVB.N mice share the H2^q^ haplotype with none of the targets. However, the patterns of susceptibility of the PSCs to LAK cells of the different strains are not explainable by a partial inhibition of NK cell activity by self-MHC class I molecules.

**Figure 2:**
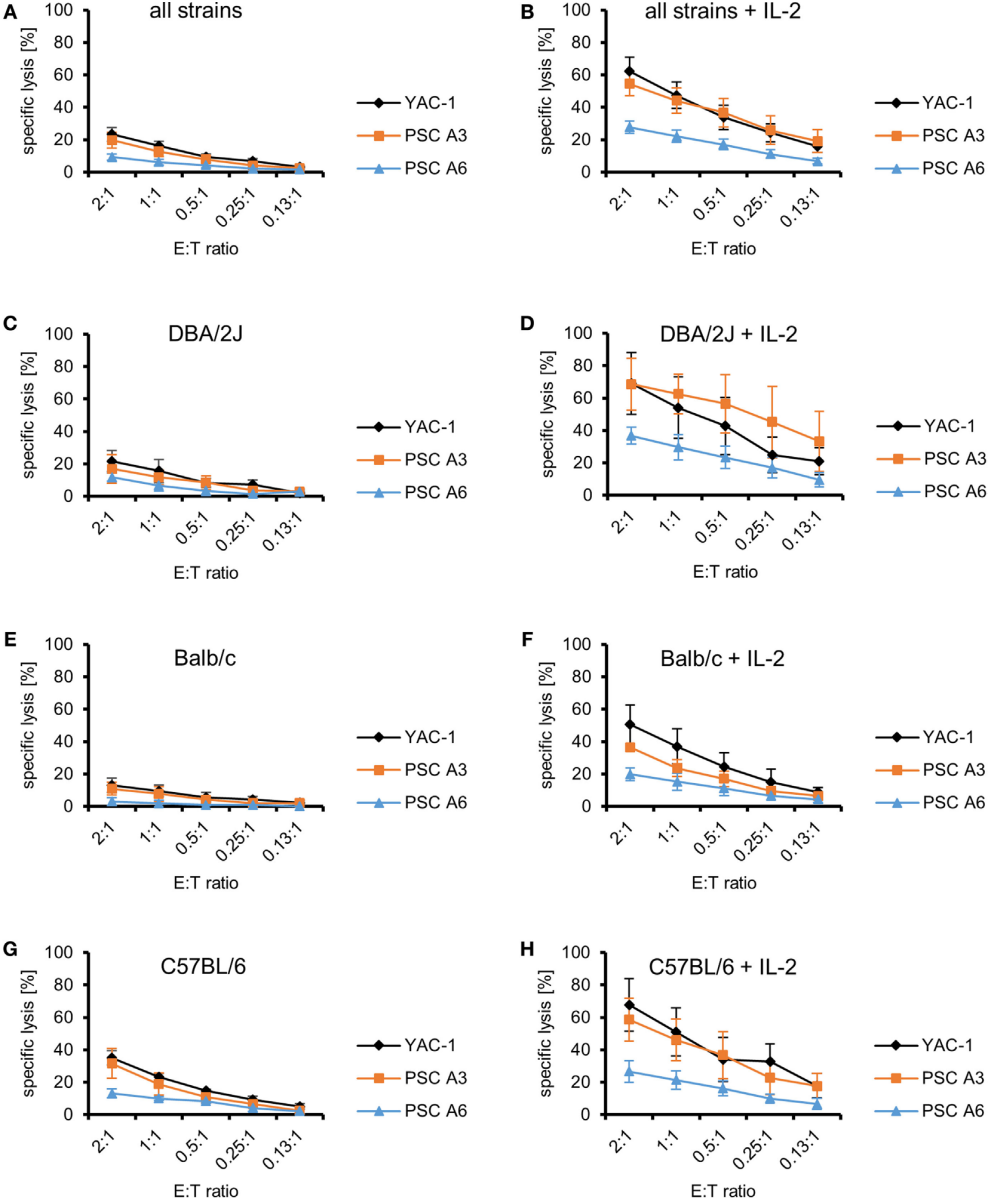
Comparison of the killing of parthenogenetic stem cell (PSC) A3, PSC A6, and YAC-1 target cells by resting and IL-2-activated natural killer (NK) cells of three mouse strains. **(A)** A summary of means of specific lysis and the SEM is shown as determined by ^51^Cr-release assays of the three target cell lines YAC-1 (*n* = 9), PSC A3 (*n* = 9), and PSC A6 (*n* = 9) by resting NK cells from three mouse strains (DBA/2J, *n* = 3; Balb/c, *n* = 3; C57BL/6, *n* = 3). **(B)** A summary of means of specific lysis and the SEM is shown as determined by ^51^Cr-release assays of the three target cell lines YAC-1 (*n* = 9), PSC A3 (*n* = 9), and PSC A6 (*n* = 9) by IL-2-activated NK cells from three mouse strains (DBA/2J, *n* = 3; Balb/c, *n* = 3; C57BL/6, *n* = 3). **(C,D)** Summary of means of specific lysis and the SEM by resting **(C)** or IL-2-activated NK cells **(D)** derived from DBA/2J mice (*n* = 3) of YAC-1 (*n* = 3), PSC A3 (*n* = 3), and PSC A6 cells (*n* = 3). **(E,F)** Summary of means of specific lysis and the SEM by resting **(E)** or IL-2-activated NK cells **(F)** derived from Balb/c mice (*n* = 3) of YAC-1 (*n* = 3), PSC A3 (*n* = 3), and PSC A6 cells (*n* = 3). **(G,H)** Summary of means of specific lysis and the SEM by resting **(G)** or IL-2-activated NK cells **(H)** derived from C57BL/6 mice (*n* = 3) of YAC-1 (*n* = 3), PSC A3 (*n* = 3), and PSC A6 cells (*n* = 3).

Next, we purified NK cells by MACS and used them as effectors against the PSC and YAC-1 target cells to confirm that indeed NK cells among LAK cells have been the cytotoxic effector cells against the PSCs. In these experiments, NK cells from the two parental strains of the PSC lines, C57BL/6 and DBA/2J, were used. In addition, NK cells from Balb/c mice were included because PSC A3 cells had formed teratomas not only in DBA/2J but also in Balb/c mice, whereas they were rejected in C57BL/6 mice in our previous study (31). Purified resting NK cells failed to kill the PSC lines efficiently at low E:T ratios (Figure 2A). Even at this low level of killing, the specific lysis of PSC A3, PSC A6, and YAC-1 target cells varied (*P* < 0.0001, ANOVA) and PSC A6 cells were more resistant than PSC A3 and YAC-1 cells (*P* < 0.01, Bonferroni *post hoc* tests). Moreover, also the cytotoxic activity of NK cells from the three strains was different (*P* < 0.0001, ANOVA; with C57BL/6 NK cells > DBA/2J NK cells > Balb/c NK cells, *P* < 0.05, Bonferroni *post hoc* tests). In a stratified analysis with respect to the NK cell donor strains, the specific lysis of the two PSC lines A3 and A6 by resting NK cells from DBA/2J mice did not differ (Figure 2C). However, PSC A3 cells were more susceptible than PSC A6 cells to killing by Balb/c (*P* = 0.0135) (Figure 2E) and C57BL/6 NK cells (*P* = 0.020, ANOVA) (Figure 2G).

After activation with IL-2 for 4 days, the NK cells, similarly as LAK cells, killed all target cell lines rather efficiently. The killing of all target cells increased significantly (YAC-1 *P* < 0.001, PSC A3 *P* < 0.0001, PSC A6 *P* < 0.0001, ANOVA) (Figures 2A,B). Increased killing by IL-2-activated NK cells was also observed for all target cell lines in separate analyses for the three mouse strains (*P* < 0.001, ANOVA). The specific lysis of PSC A3, PSC A6, and YAC-1 cells by IL-2-activated NK cells was different (*P* < 0.0001, ANOVA). Overall, PSC A6 cells were more resistant than PSC A3 cells (*P* < 0.0001) and YAC-1 cells (*P* < 0.0001, Bonferroni *post hoc* test), whereas the specific lysis of PSC A3 cells was in similar range as YAC-1 control cells and not significantly different (Figure 2B). IL-2-activated NK cells from DBA/2J (Figure 2D), Balb/c (Figure 2F), and C57BL/6 mice (Figure 2H) differed in their efficacy to kill the three target cell lines (*P* < 0.0001, ANOVA). The NK cells did not significantly vary in their ability to kill YAC-1 cells (*P* = 0.144, ANOVA) (Supplementary Figure 3). However, they differed in killing of PSC A3 (*P* = 0.0006) and PSC A6 cells (*P* = 0.004, ANOVA) (Figures 2D,F,H). DBA/2J NK cells were more efficient than Balb/c NK cells (PSC A3: *P* = 0.0004; PSC A6: *P* = 0.0028, Bonferroni *post hoc* tests). The PSC A3 cells were lysed more efficiently than PSC A6 cells by NK cells from DBA/2J (*P* = 0.0011) and C57BL/6 mice (*P* = 0.0049, ANOVA). In the experiments with Balb/c NK cells, a trend toward this difference was observed (*P* = 0.0695). Thus, NK cells derived from mice carrying the H2^d^ haplotype (DBA/2J and Balb/c) were not inhibited by the PSCs. Although, the C57BL/6 NK cells were killing the PSC A3 cells (H2^d^) better than the PSC A6 cells (H2^b/d^) this does not suggest a specific inhibition by MHC class I molecules of the H2^b^ haplotype as this pattern was observed also with NK cells of the other strains.

**Figure 3:**
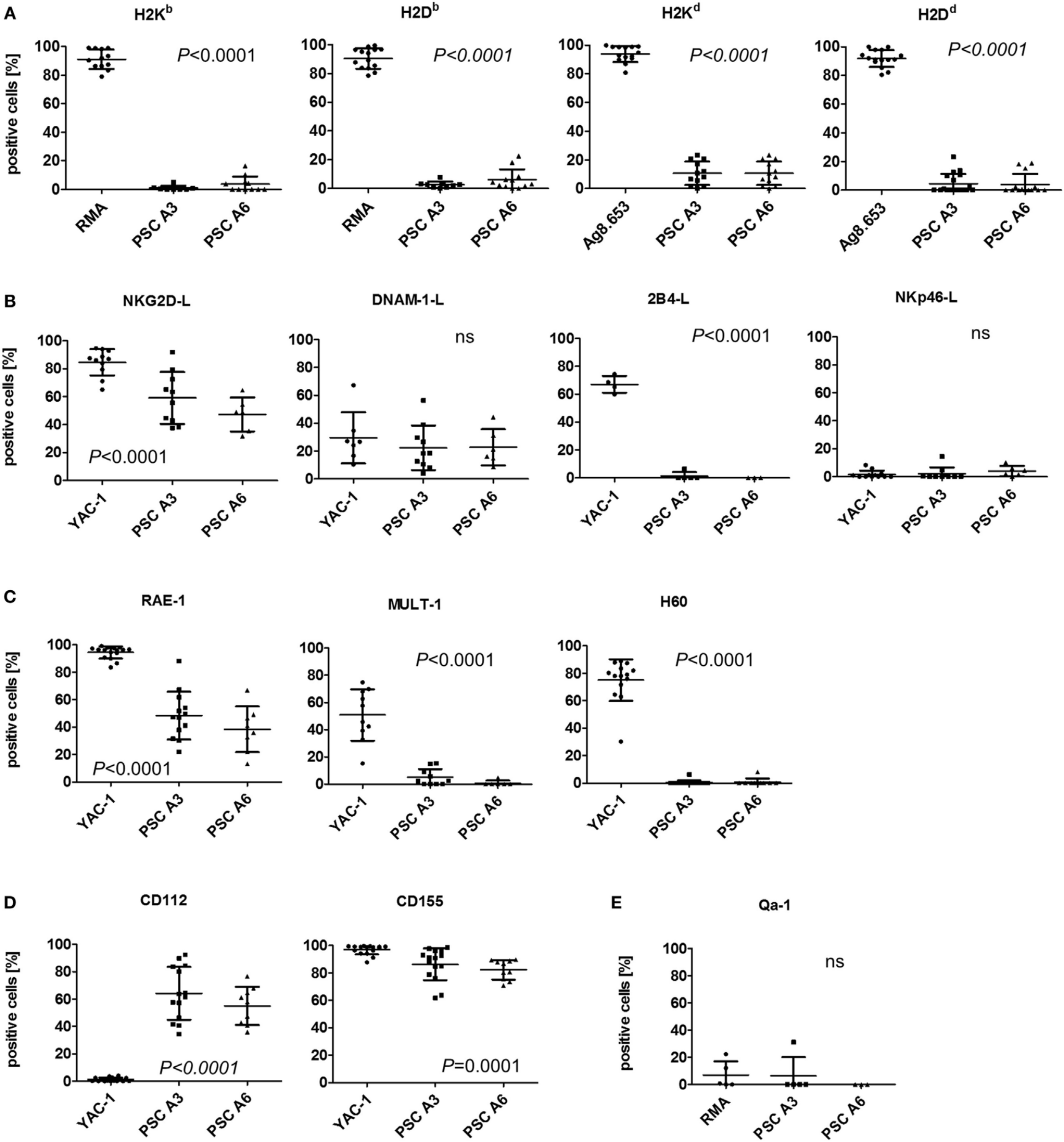
Analysis of the expression of natural killer (NK) receptor ligands on parthenogenetic stem cell (PSC) A3, PSC A6, and control cells. The percentage of cells expressing the indicated NK receptor ligands is shown as determined by flow cytometry in 3–18 individual experiments. In addition, the mean ± SD is indicated. Differences between the three cell lines were analyzed by Kruskal–Wallis tests and the respective *P*-values or non-significant (ns) results are indicated. **(A)** The expression of classical major histocompatibility complex (MHC) class I molecules, which can serve as ligands for inhibitory NK receptors has been determined by allotype-specific monoclonal antibodies. RMA (H2^b^) or Ag8.653 (H2^d^) cells served as positive controls. **(B)** The expression of ligands of the activating NK receptors NKG2D, DNAM-1, 2B4, and NKp46 on the PSCs in comparison to YAC-1 cells has been tested by binding of recombinant receptor-Fc fusion proteins. **(C)** The presence of the NKG2D receptor ligands RAE-1, MULT-1, and H60 has been analyzed in comparison to YAC-1 cells. **(D)** The expression of DNAM-1 receptor ligands CD112 and CD155 has been tested in comparison to YAC-1 cells. **(E)** The expression of the non-classical MHC class I molecule Qa-1, which is a murine functional homolog of human leukocyte antigen-E has been determined in comparison to RMA cells.

The cytotoxic activity of NK cells is controlled by inhibitory and activating NK receptors. Ligands for inhibitory NK receptors are mainly MHC class I molecules. Murine pluripotent stem cells, including ESCs, iPSCs, and maGSCs, have been shown to lack MHC class I molecules at least at a level detectable by flow cytometry (39). Both PSC lines were also largely lacking H2K and H2D molecules, as exemplified in Figure S4 in Supplementary Material, although especially H2K^d^ molecules were found at low levels on average on 10.6% of the PSC A3 and on 7.0% of the PSC A6 cells (Figure 3A). RMA (H2^b^) or Ag8.653 (H2^d^) served as positive control cells in these experiments. Recombinant Fc fusion proteins of the activating NK receptors NKG2D, DNAM-1, 2B4, and NKp46 were used to detect the respective ligands on the PSCs and YAC-1 cells (Figure 3B). Both PSC lines expressed ligands for the activating NK receptors NKG2D and DNAM-1 as demonstrated by binding of recombinant receptor proteins. Ligands for the 2B4 and NKp46 receptors were not detected. The NKG2D ligands present on the PSC lines belonged to the RAE-1 family (Figure 3C). MULT-1 and H60 proteins, by contrast, were hardly expressed. Both DNAM-1 ligands, CD112 and CD155, were found on the PSC lines (Figure 3D). However, the PSC line A6 largely lacked the functional HLA-E homolog Qa-1^b^ (Figure 3E). For most molecules tested by flow cytometry (with exception of DNAM-1 and NKp46 ligands as well as Qa-1 molecules), significant expression differences between the analyzed cells were found (ANOVA, see Figures 3A–E). However, the *post hoc* tests indicated differences only between the control cells and the PSC lines but none between the two PSC lines. In addition to the percentage of positive cells for the various molecules, their MFI has been determined and showed the same patterns (Figure S5 in Supplementary Material). In summary, the PSC lines express ligands for activating NK receptors and largely lack MHC class I molecules that serve as ligands for inhibitory receptors. Consequently, they are targets for LAK and NK cells.

At the mRNA level, *H2K* and *B2m* as well as transcripts of the chaperones involved in antigen presentation (*Calr, Canx, Erp57*) were found in the PSC lines although they were mostly less abundant than in RMA control cells or DBA/2J splenocytes (Figure S6A in Supplementary Material). *Tap1* and *Tapbp* mRNAs were found at low level in the PSC lines. *H2D, Tap2, Qa1b*, and the genes *Psmb9* and *Psmb8* encoding the alternative subunits of the immunoproteasome (LMP2 and LMP7), by contrast, were hardly expressed in the PSCs. Transcripts of *Nectin2* (encoding CD112), *Pvr* (encoding CD155), as well as *Raet1* (encoding RAE-1) and at lower levels *Ulpb1* (encoding MULT-1) were detected in PSCs in contrast to *H60* mRNA. Interestingly, transcripts of the NK receptor genes *Cd226* (encoding DNAM-1) and *Klrk1* (encoding NKG2D) were detectable at very low level. The genes encoding the co-stimulatory molecules CD80 and CD86 were basically not expressed in the PSC lines.

### Differentiation of PSCs Reduces the Susceptibility to NK Cells

Differentiated cells are expected to be able to inhibit at least autologous NK cells to ensure self-tolerance. Therefore, we differentiated the PSC lines *in vitro* for 12 days in an undirected manner and determined the susceptibility of the differentiated cells to LAK cells in ^51^Cr-release assays. The differentiation significantly reduced the expression of the stem cell marker SSEA-1 indicating that the *in vitro* differentiation process was successful (Figures S7A–C in Supplementary Material).

**Figure 4:**
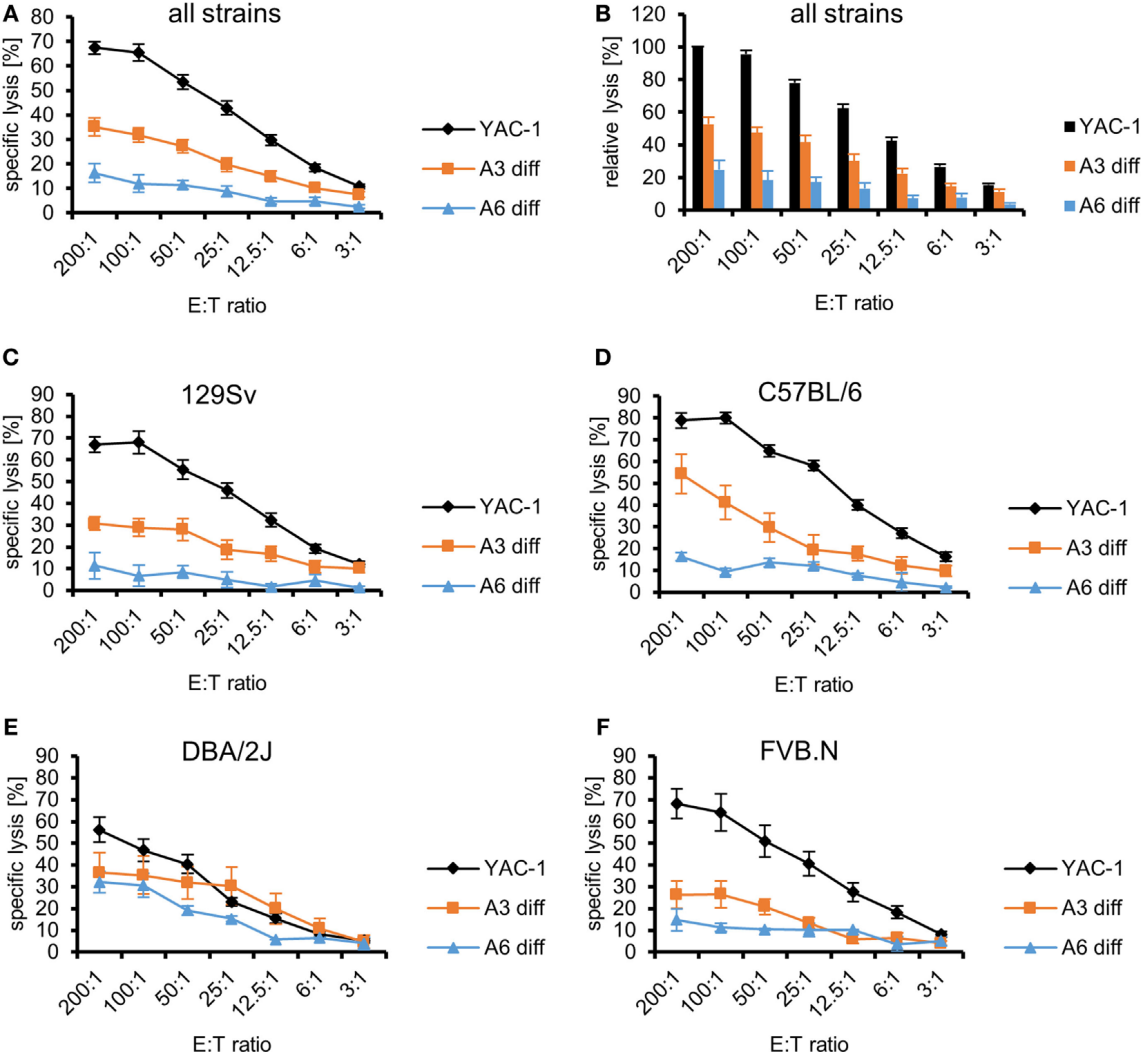
Comparison of the killing of *in vitro*-differentiated A3 and A6 cells and YAC-1 control cells by lymphokine-activated killer (LAK) cells of four mouse strains. **(A)** A summary of means of specific lysis and SEM is shown as determined by ^51^Cr-release assays of the three target cell lines YAC-1 (*n* = 28), parthenogenetic stem cell (PSC) A3 (*n* = 16), and PSC A6 (*n* = 12) using LAK cells from four mouse strains (129Sv, *n* = 12; C57BL/6, *n* = 5; DBA/2J, *n* = 5; FVB.N, *n* = 6). **(B)** The data are shown as relative lysis calculated by setting the specific lysis of YAC-1 cells at the highest effector to target (E:T) ratio (200:1) in individual experiments to 100% and adjusting the relative lysis at lower E:T ratios and of other target cells accordingly. **(C)** Summary of means of specific lysis and SEM by LAK cells derived from 129Sv mice (*n* = 12) of YAC-1 (*n* = 12), differentiated A3 (*n* = 6), and differentiated A6 cells (*n* = 6). **(D)** Summary of means of specific lysis and SEM by LAK cells derived from C57BL/6 mice (*n* = 5) of YAC-1 (*n* = 5), differentiated A3 (*n* = 3), and differentiated A6 cells (*n* = 2). **(E)** Summary of means of specific lysis and SEM by LAK cells derived from DBA/2J mice (*n* = 5) of YAC-1 (*n* = 5), differentiated A3 (*n* = 3), and differentiated A6 cells (*n* = 2). **(F)** Summary of means of specific lysis and SEM by LAK cells derived from FVB.N mice (*n* = 6) of YAC-1 (*n* = 6), differentiated A3 (*n* = 4), and differentiated A6 cells (*n* = 2).

The differentiated cells and YAC-1 cells were used as targets for LAK cells derived from the same four strains that were used in the experiments with undifferentiated PSCs. The specific lysis of the target cells (differentiated A3, differentiated A6, and YAC-1 cells) by LAK cell from all strains varied (*P* < 0.0001, ANOVA). Both differentiated cell types A3 (*P* < 0.0001) and A6 (*P* < 0.0001) were more resistant than YAC-1 cells and A6 cells were more resistant than A3 cells (*P* < 0.0001) as indicated by Bonferroni *post hoc* testing (Figure 4A). The relative lysis of the targets is illustrated in Figure 4B to allow for comparison with previously published data of other pluripotent stem cell types (36). In addition, LAK cells derived from the four strains (129Sv, C57BL/6, DBA/2J, and FVB.N) varied in their efficacy to kill the targets (*P* < 0.0001, ANOVA) (Figures 4C–F). LAK cells from C57BL/6 mice were more potent than the others (*P* < 0.001, Bonferroni *post hoc* tests). When comparing the specific lysis of the two differentiated cell types only, C57BL/6 and DBA/2J LAK cells were more efficient killers than LAK cells derived from 129Sv and FVB.N mice (*P* < 0.001, Bonferroni *post hoc* tests). Notably, the specific lysis of the two differentiated target cell lines A3 and A6 was significantly different when effector cells were used derived from 129Sv (*P* < 0.0001, ANOVA) and C57BL/6 (*P* = 0.0017) but not DBA/2J (*P* = 0.1182) or FVB.N mice (*P* = 0.0989). This could indicate that differentiated A6 cells expressing H2^b^ MHC class I molecules better inhibit NK cells of the haplotype H2^b^ (129Sv and C57BL/6) than the A3 cells carrying the H2^d^ haplotype only. However, A6 cells did not efficiently inhibit NK cells from DBA/2J (H2^d^) mice. The relative lysis of the targets by effector cells of the four strains is shown in Figures S8A–D in Supplementary Material.

As expected, differentiated cells were overall more resistant to LAK cells from all strains than undifferentiated PSCs (*P* < 0.0001, ANOVA) (Figures S9A in Supplementary Material). The PSC A3 cells were more susceptible to killing than PSC A6 cells (*P* < 0.0001, Bonferroni *post hoc* tests), differentiated A3 cells (*P* < 0.0001), and differentiated A6 cells (*P* < 0.0001). Differentiated A6 cells were significantly more resistant than all other targets (*P* < 0.0001, Bonferroni *post hoc* tests). However, the specific lysis of PSC A6 cells and differentiated A3 cells did not differ significantly. This pattern was also observed (*P* < 0.0001, Bonferroni *post hoc* tests) when the specific lysis elicited by 129Sv or FVB.N LAK cells was analyzed separately (Figures S9B,E in Supplementary Material). When the killing elicited by LAK cells derived from C57BL/6 or DBA/2J was separately analyzed, only differentiated A6 cells were more resistant than the other cell types (Figures S9C,D in Supplementary Material). Overall, both differentiated A3 (*P* < 0.0001) and differentiated A6 cells (*P* < 0.0001, ANOVA) were more resistant to LAK cells from all strains than the respective undifferentiated PSCs. Both differentiated cell types A3 (*P* < 0.0001) and A6 (*P* < 0.0001, ANOVA) were more resistant than the respective undifferentiated PSCs to 129Sv LAK cells (Figure S9B in Supplementary Material). The same result was observed for FVB.N effectors (A3: *P* < 0.0001; A6: *P* = 0.0308) (Figure S9E in Supplementary Material). When C57BL/6 or DBA/2J effector cells were used, only the differentiation of A6 cells decreased the susceptibility to LAK cells (C57BL/6: *P* = 0.0002; DBA/2J: *P* = 0.0216) (Figures S9C,D in Supplementary Material). The killing of PSC A3 and differentiated A3 cells, by contrast, was not significantly different (C57BL/6: *P* = 0.6651; DBA/2J: *P* = 0.1216).

**Figure 5:**
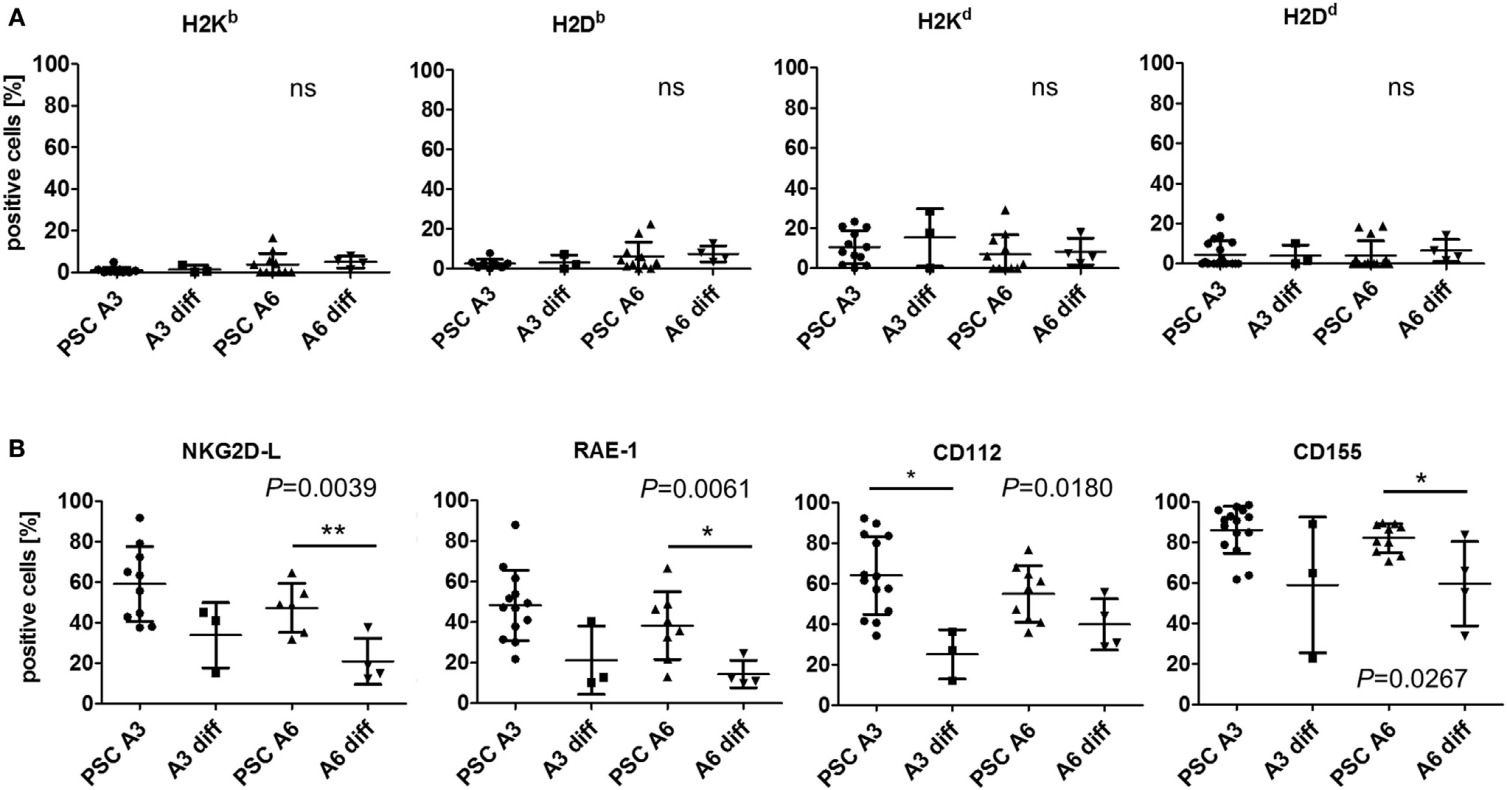
Comparison of the expression of major histocompatibility complex (MHC) class I molecules and other natural killer (NK) receptor ligands on parthenogenetic stem cell (PSC) A3, PSC A6, and *in vitro*-differentiated cells. The percentage of cells expressing the indicated NK receptor ligands is shown as determined by flow cytometry in 3–18 individual experiments. In addition, the mean ± SD is indicated. Differences between the cells were analyzed by Kruskal–Wallis tests and the respective *P*-values or non-significant (ns) results are given in the figure. Significant differences in *post hoc* tests between PSCs and the respective differentiated cells are indicated (**P < 0.01, * *P* < 0.05). **(A)** The expression of classical MHC class I molecules has been determined by allospecific monoclonal antibodies (mAbs). **(B)** The expression of ligands of the activating NK receptors NKG2D (NKG2D-L) has been determined by binding of the recombinant receptor-Fc fusion protein. Expression of the specific NKG2D ligand RAE-1 and the DNAM-1 ligands CD112 and CD155 has been analyzed by mAbs.

At the mRNA level, the differentiation of the PSCs partly increased the expression of MHC class I transcripts, such as *H2D* and *H2K*, in A6 cells (Figure S6A in Supplementary Material). However, expression of most genes encoding proteins involved in antigen expression was not markedly increased or even decreased. Notably, transcripts of most NKG2D and DNAM-1 ligands were less abundant in both differentiated cell types. At the protein level, the differentiated cells also largely lacked MHC class I molecules as determined by flow cytometry and no significant differences to the PSCs were detected (Figure 5A). However, NKG2D ligands and specifically RAE-1 as well as the DNAM-1 ligands CD112 and CD155 were differently expressed and downregulated on differentiated cells although not always all molecules were downregulated on both cell types (Figure 5B).

### PSCs Are Largely Resistant to Cytotoxic T Cells

**Figure 6:**
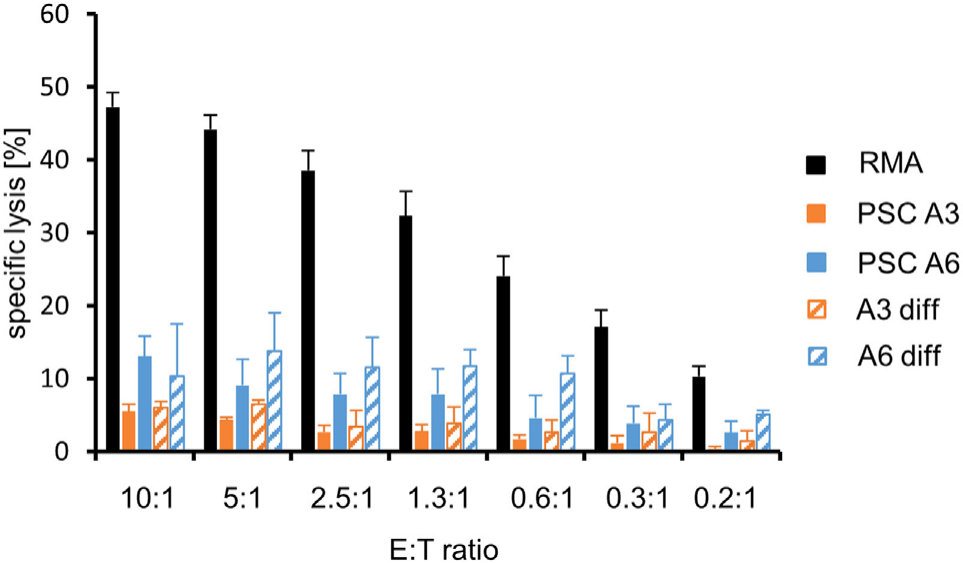
Comparison of the killing of parthenogenetic stem cell (PSC) A3, PSC A6, and *in vitro*-differentiated cells as well as RMA control cells by peptide-specific cytotoxic T lymphocytes from OT-I mice. A summary of means of specific lysis and SEM is shown as determined by ^51^Cr-release assays of the target cells RMA (*n* = 13), PSC A3 (*n* = 4), PSC A6 (*n* = 4), differentiated A3 cells (*n* = 2), and differentiated A6 cells (*n* = 3). The killing has been assayed after pulsing of the target cells with the appropriated peptide SIINFEKL.

In previous experiments, several murine pluripotent stem cell types, including ESCs, iPSCs, and maGSCs, were targets for peptide-specific CTLs derived from TCR-transgenic OT-I mice after pulsing with the appropriate peptide SIINFEKL despite being negative for MHC class I molecules in flow cytometry (39). Of the two PSC cell lines analyzed here, only one, i.e., A6, carried the required MHC molecule H2K^b^. Thus, the line A3 carrying the H2^d^ haplotype served as negative control, whereas RMA cells (H2^b^) were used as positive control. The specific lysis of the three target cell lines (RMA, PSC A3, and PSC A6) varied (*P* < 0.0001, ANOVA). However, compared to the positive control target cell line RMA, both PSC lines were largely resistant to the CTLs. The PSC A3 target cells showed a minimal background killing of 5% at the highest E:T ratio but the specific lysis of PSC A6 targets was only slightly higher with 13% on average at the highest E:T ratio (Figure 6). The low killing of PSC A6 cells and differentiated A6 cells was peptide and Ca^2+^-dependent (Figure S10 in Supplementary Material), indicating a lysis by CTLs using the granule exocytosis pathway of killing that involves perforin and granzymes. *Post hoc* tests indicated that the specific lysis of both PSC lines did differ significantly from RMA cells (*P* < 0.0001). Moreover, PSC A6 cells were also more susceptible to the peptide-specific CTLs than PSC A3 cells (*P* < 0.05). The *in vitro* differentiation of PSC A6 cells over 12 days was not sufficient to significantly increase the susceptibility of the cells to CTLs (*P* = 0.066) (Figure 6). At the mRNA level, we did not observe a strong expression of *Ido1, Arg1*, or *Tgfb1*, which encode proteins that have a role in the suppression of CTL activity, neither in the PSCs nor in the differentiated cells. Similarly, *Serpinb9* that encodes a granzyme B inhibitor was not strongly expressed in any of the CTL-resistant targets (Figure S6B in Supplementary Material). However, the CTL resistance is in agreement with the low expression of H2K^b^ that was found on the differentiated cells (see Figure 6). Thus, the differentiation protocol applied here does not result in a cell population with a normal susceptibility to CTL-mediated killing.

## Discussion

In the last few years, first clinical trials have been set up to investigate the therapeutic potential of grafts derived from pluripotent stem cells to treat degenerative diseases, including macular degeneration, spinal cord injury, and heart failure (3, 4). Most of these trials use human ESCs to *in vitro* differentiate the grafts which are transplanted. However, the pluripotent stem cell type best suited for therapeutic purposes is still debated (43). Although autologous patient-specific iPSCs can be routinely generated, they might have disadvantages with respect to their genetic integrity (19) and the complex processes to generate autologous grafts are expected to be time consuming and expensive. Therefore, allogeneic transplantations of grafts derived from banked HLA-matched pluripotent stem cell lines (4, 28, 29) might be a more feasible alternative (44). In this respect, PSCs are an interesting alternative stem cell type since their immunogenetic complexity is reduced due to their usually homologous genotype particularly in the MHC locus. However, the immunological properties of PSCs had hardly been analyzed previously and, therefore, it was unclear whether and to which extent they might differ from other pluripotent stem cell types.

We have previously reported that mouse ESC, maGSC, and iPSC lines are highly susceptible targets for LAK cells as well as purified IL-2-activated NK cells (32, 36, 37). These pluripotent stem cell types were all killed by allogeneic and syngeneic LAK cells to a similar extent or even better than YAC-1 cells that are the typical target cell line for murine NK cells. However, individual stem cell lines varied in susceptibility to the effector cells and effector cells from different strains varied in their efficacy against specific targets. We have now demonstrated that also PSC lines are killed by syngeneic and allogeneic LAK and NK cells with a similar variation with respect to individual cell lines and donor strains of the effector cells. NK cell cytotoxicity *in vivo* is tightly controlled and an education or licensing process ensures that only those NK cells acquire responsiveness, which can receive inhibitory signals by self-MHC molecules (45). NK cells may react against cells expressing non-self MHC molecules if these fail to engage the inhibitory NK cell receptors of these NK cells. The PSC A6 cells carry, in addition to the MHC haplotype H2^d^, a further haplotype (H2^b^) that is not present in PSC A3 cells (H2^d/d^). However, the patterns of more or less lysis by LAK or NK cells from the different donor strains was not explainable by a stronger or weaker engagement of inhibitory NK receptors by self-MHC molecules on PSCs. This result is in agreement with our finding that the PSC lines, similarly to murine ESCs, maGSCs, and iPSCs (32, 36, 38, 39), largely lack MHC class I molecules at the plasma membrane as determined by flow cytometry. Instead, the PSC lines expressed ligands of activating NK receptors also similarly as the other pluripotent stem cell types (32, 36, 38). The cytotoxic activity of NK cells toward target cells is controlled by the balance of signals from inhibitory and activating NK receptors (46). On the PSCs, NKG2D ligands of the RAE-1 family as well as the DNAM-1 ligands CD112 and CD155 were found. The combination of a lack of MHC class I molecules and the presence of ligands of activating NK receptors can explain the susceptibility of PSCs and other pluripotent stem cell types to NK cells. However, the expression patterns of these NK receptor ligands cannot explain the higher susceptibility of PSC A3 than PSC A6 cells to LAK and NK cells that we observed in our experiments. The functional relevance of the NKG2D receptor for the killing of ESCs (32, 37, 47) and other pluripotent stem cell types (36, 37) has been demonstrated in inhibition and gene knockout experiments. Initially, some studies reported resistance of ESCs to NK cells (48, 49), while we and others found them to be highly susceptible (32, 36, 47, 50). The conflicting results appear to be explainable mainly by the activation status of NK cells. We have shown here that PSCs were largely resistant to resting but susceptible to IL-2-activated NK cells from several mouse strains. This phenotype is similar to other pluripotent stem cell types, including ESCs, iPSCs, and maGSCs (37). Moreover, we found previously that the transplantation of maGSCs into the myocardium of mice by open heart surgery activated NK cells and significantly increased their ability to kill maGSCs in direct *ex vivo* assays (38). It has also been reported that human cardiac-derived stem/progenitor cells were resistant to resting allogeneic NK cells but moderately triggered killing by cytokine-activated NK cells (51). Moreover, we have recently shown that human iPSCs were largely resistant to resting NK cells but readily killed by IL-2-activated syngeneic and allogeneic NK cells. Notably, the human iPSCs expressed also similar patterns of ligands for activating NK receptors as the murine pluripotent stem cells (52). However, human iPSCs express higher amounts of MHC class I molecules than murine iPSCs (39, 52) and a higher expression of MHC class I molecules has been also found on human ESCs (53). In view of these data, one might conclude that a high susceptibility to activated NK cells constitutes a hallmark of pluripotent cells.

The undirected *in vitro* differentiation of the PSC lines reduced the susceptibility of the differentiated cells to LAK effectors. However, the degree of resistance varied for the two cell lines and also the effector cells of the different donor strains varied in their efficacy to kill differentiated target cells. The acquired resistance was accompanied by a downregulation of the ligands for activating NK receptors but not by an upregulation of MHC class I molecules. These patterns closely resemble the patterns that we observed previously on other murine pluripotent stem cell types, including ESCs, iPSCs, and maGSCs, which also acquired partial resistance to LAK cells upon *in vitro* differentiation (36). A longer differentiation of ESCs either *in vitro* or *in vivo* can lead to an upregulation of MHC class I molecules (54–57), which is expected to further increase the resistance of the differentiated cells to NK cells. In view of these phenotypes, NK cells could be an interesting player after transplantation of stem cell-derived grafts because they can specifically target undifferentiated cells, which are at risk to elicit tumors in the recipients (18). NK cells that can kill undifferentiated cells, which might contaminate grafts in trace amounts, could increase the safety of transplantations (38, 58) largely independent of the pluripotent stem cell type that is used for the generation of grafts. However, individual differences between stem cell lines and variations in the efficacy of the NK cells as well as immunosuppressive treatments applied after transplantation might modulate the threshold of undifferentiated cells that could be tolerated in a graft (52).

Notably, the PSC line A6, which carries the H2^b^ haplotype has been largely resistant to killing executed by peptide-specific CTLs derived from OT-I mice, which recognize the peptide SIINFEKL in a H2K^b^-restricted manner (40). Previously, we have determined the susceptibility of other pluripotent stem cell lines to these CTLs (39). Two ESC, three maGSC, and one iPSC line have been all lysed to a greater extent than PSC A6 targets. The relative lysis of these stem cell lines has been in the range of 40–80% compared to RMA control cells at an E:T ratio of 10:1 although the expression of H2K^b^ molecules on these targets was also mostly not detectable by flow cytometry (39). However, it is known that extremely low numbers of MHC/peptide complexes on target cells are sufficient to elicit killing by activated CTLs (59–61). Similarly to the other pluripotent stem cell types that we investigated previously (39), the PSC lines expressed some MHC class I genes, such as *H2K, B2m*, and the chaperones involved in the assembly of MHC class I complexes (*Calr, Canx*, and *Erp57*), but *Tap2*, the gene encoding the transporter associated with antigen expression 2 was less expressed. Potentially, the low expression of the *Tap2* gene could be responsible for the low expression of MHC class I molecules on pluripotent stem cells. Notably, the killing of differentiated A6 cells by CTLs increased slightly compared to the undifferentiated cells, although the H2K^b^ expression did not increase at a level detectable by flow cytometry. It has been previously reported that Serpin-6, encoded by the *Serpinb9* gene, protects ESCs from killing by CTLs using the granule exocytosis pathway to kill target cells (62). However, in the PSC lines as well as in the differentiated cells, *Serbinb9* mRNA was present only in trace amounts. It has also been reported that human ESCs suppress T cell responses by an arginase 1-dependent mechanism (63). Arginase 1 depletes l-arginine similarly as the indoleamine 2,3-dioxygenase catabolizes tryptophan (64). The lack of these amino acids is known to inhibit T cell function (65). In the PSCs as well as in the differentiated cells, *Arg1* and *Ido1* were hardly or weakly expressed. Moreover, it has been reported that murine ESCs inhibit T cells by secretion of the transforming growth factor β1 (35). Again, the *Tgfb1* gene was hardly expressed neither in the PSCs nor in the differentiated cells. Thus, the mechanism behind the low killing of PSCA6 cells and differentiated A6 cells by the CTLs remains to be elucidated. More PSC lines need to be analyzed to determine whether the low susceptibility of PSC A6 cells to killing by CTLs is a specific feature of this cell line or a general feature of PSCs when compared to other pluripotent stem cell types. However, *in vivo* PSC A6 cells have been rejected in most allogeneic recipients despite this more CTL-resistant phenotype of *in vitro*-differentiated cells (31).

## Conclusion

Parthenogenetic stem cells are a therapeutically interesting pluripotent stem cell type due to their reduced immunogenetic complexity that would make the selection of MHC-matched stem cells for the generation of grafts easier feasible. Despite this specific feature, the immunological properties of PSCs have previously not been characterized. We have shown now that the immunological properties of PSCs are in many respects similar to other pluripotent stem cell types. PSCs, as other murine pluripotent stem cell types, largely lack MHC class I molecules but express ligands for activating NK receptors, including RAE-1, CD112, and CD155 and are, consequently, susceptible to killing by cytokine-activated NK cells. This phenotype appears to constitute a general feature of pluripotent cells. Therefore, the activity of NK cells potentially increases the safety of transplantations of pluripotent stem cell-derived grafts, which may contain traces of undifferentiated cells that could be tumorigenic in the recipients. PSC-derived differentiated cells acquired resistance to NK cell-mediated killing although variations with respect to the PSC line and the NK cell donors have been observed. Interestingly, the PSC line A6 and *in vitro*-differentiated A6 cells were rather resistant to CTL-mediated killing. It is of interest to determine the immunological properties of specific PSC-derived cell types with therapeutic relevance such as cardiomyocytes, neurons, or hepatocytes.

## Ethics Statement

For this study, mice were narcotized and killed before spleens were taken for *in vitro* experiments. No specific permission for animal experimentation was required to obtain the organs from the sacrificed mice for the *in vitro* experiments performed in this study according to German law. The University Medical Center Göttingen has appointed a commissioner who ensured that the mice were scarified in accordance with the German law and European directives (2010/63/EU). The number of mice used to obtain organs has been reported to the local government (Lower Saxony State Office for Consumer Protection and Food Safety) in accordance with legal requirements.

## Author Contributions

RD, MD, and W-HZ designed the study; HJ, VM, CG, SM, and LE acquired data; MD and W-HZ provided reagents, HJ, VM, CG, SM, MD, and RD analyzed and interpreted data; RD wrote the manuscript; all authors edited the draft and approved the final version of the manuscript.

## Conflict of Interest Statement

The authors declare that the research was conducted in the absence of any commercial or financial relationships that could be construed as a potential conflict of interest. The reviewer NJ and handling Editor declared their shared affiliation, and the handling Editor states that the process nevertheless met the standards of a fair and objective review.
