# Supplementary material for: Immunological Properties of Murine Parthenogenetic Stem Cells and Their Differentiation Products
Source: Front Immunol. 2017 Aug 4;8:924. doi: 10.3389/fimmu.2017.00924 (PMC5543037; doi:10.3389/fimmu.2017.00924)
Supplement: Supplementary file 1 [file presentation_1.pdf]

Supplementary Material

Immunological properties of murine parthenogenetic stem cells and their differentiation products

Hannah Johannsen, VijayakumarMuppala, Carina Gröschel, Sebastian Monecke, Leslie Elsner, Michael Didié, Wolfram-Hubertus Zimmermann, Ralf Dressel

- Supplementary Tables 1 and 2
- Supplementary Figures 1 to 10

Supplementary Table 1. Antibodies and isotype controls used for flow cytometry

Antigen	Isotype	Clone	Label	Supplier
CD3	rat IgG_{2b}	17A2	FITC	BioLegend, Fell, Germany
CD49b	rat IgM	DX5	PE	BioLegend, Fell, Germany
CD112	rat IgG_{2a}	502-57	-	Santa Cruz, Heidelberg, Germany
CD155	rat IgG_{2a}	TX56	-	BioLegend, Fell, Germany
RAE-1	rat IgG_{2a}	186107	-	R&D Systems, Wiesbaden, Germany
$H2K^b$	mouse IgG_{2a}	AF6-885	PE	BioLegend, Fell, Germany
$H2D^b$	mouse IgG_{2b}	KH95	PE	BioLegend, Fell, Germany
$H2K^d$	mouse IgG_{2a}	SF 1-1.1	PE	BioLegend, Fell, Germany
$H2D^d$	mouse IgG_{2a}	34-2-12	PE	BioLegend, Fell, Germany
$Qa\text{-}1^b$	mouse IgG_1	6A8.6F10.1A6	Biotin	BD Bioscienes, Heidelberg, Germany
SSEA-1	mouse IgM	MC-480	-	BioLegend, Fell, Germany
mouse IgG	goat IgG	polyclonal (155-095-062)	FITC	Jackson Laboratories, via Dianova, Hamburg, Germany
Mouse IgM	goat IgG	polyclonal (155-095-020)	FITC	Jackson Laboratories, via Dianova, Hamburg, Germany
rat IgG	goat IgG	polyclonal (112-095-062)	FITC	Jackson Laboratories, via Dianova, Hamburg, Germany
human IgG	goat IgG	polyclonal (109-095-098)	FITC	Jackson Laboratories, via Dianova, Hamburg, Germany
-	rat IgM	RTK2118	PE	BioLegend, Fell, Germany
-	rat IgG_{2b}	RTK4530	FITC	BioLegend, Fell, Germany
-	mouse IgG_{2a}	MOPC-173	PE	BioLegend, Fell, Germany
-	mouse IgG_{2b}	MPC-11	PE	BioLegend, Fell, Germany

The following abbreviations are used: FITC, fluorescein isothiocyanate, and PE, phycoerythrin.

Supplementary Table 2. Primers used for qPCR

Gene	Sequence 5'-3'	Reference Sequence
Arg1	ACC TGG CCT TTG TTG ATG TCC<br>AGC ACC ACA CTG ACT CTT CCA TTC	NM_007482.3
B2m	CTC ACA CTG AAT TCA CCC CC<br>CAG TAG ACG GTC TTG GGC TC	NM_009735.3
Calr	AGC TGT TTC CGA GTG GTT TG<br>GAT CAG CAC ATT CTT GCC CTT G	NM_007591.3
Canx	TGA TCC TCT TCT GCT GTT CTG G<br>TTC ATC CTT CAC ATC TGG CTG G	NM_007597.3
Cd80	ATT GCT GCC TTG CCG TTA CAA CTC<br>GGT TCT TAT ACT CGG GCC ACA C	NM_009855.2
Cd86	TCA GTA TCT CCA ACA GCC TCT CTC<br>ACT CCG TTT CCA GAA CAC ACA C	NM_019388.3
Cd226	TCG TTG GAG GGT TAG TTT CAC<br>GCT ACC TTA CTC TGT TTA TCC CTG	NM_178687.2
Nectin2	CGA GAG TCA CCC AGC ACA G<br>TGT TGT CGG CAG ATG AGG ATG	NM_008990.3
Pvr	AGC ACG AAC ACG GGT GAC TTT C<br>CTA GGG CAT TGG TGA CTT C	NM_027514.2
Erp57	TGA TAA AGA TGC CTC AGT GGT GGG<br>TGT TGG TGT GTG CAA ATC GGT AG	NM_007952.2
H2D	CCC TGT GAG CTT GGG TTC AG<br>ACA GGG CAG TGC AGG GAT AG	NM_010380.3
H2K	CCT GGA GTG GAC TTG GTG AC<br>GGT GTA GAG GGG TGG ACT GG	NM_001001892.2
H60	GTG GCT TCT CCA GCA AAG GA<br>GCC ACC ACT CTC ATG GGT TC	NM_198193.2
Hprt1	GTC CTG TGG CCA TCT GCC TA<br>GGG ACG CAG CAA CTG ACA TT	NM_013556.2
Ido1	CCA CAC TGA GCA CGG ACG G<br>TGC GGG GCA GCA CCT TTC G	NM_008324.1
Klrk1	GCT GGT TAA GTC CTA TCA CTG G<br>TTG AGC CAT AGA CAG CAC AG	NM_033078.3
Psmb9	ATC TTC TGT GCC CTC TCA GGT TC<br>AGA TGC GCT AAC AAG TCC TCA C	NM_013585.2
Psmb8	GCT TAT GCT ACC CAC AGA GAC AAC<br>CAC TGA CAT CGG AAC TCT CCA C	NM_010724.2
Qa1b	GAT GTT GCT TTT TGC CCA C<br>TAG CCG ACA ATG ATG AAC C	NM_010398.3
Raet1	CCA AGG AGA CGC CAG AGG AG<br>CAG GAC CTC TCC AAG AAC AGC A	NM_020030.2

Gene	Sequence 5'-3'	Reference Sequence
Serpinb9	TGC AGA CAA AAC TTG TGA AGT CCT C<br>TGC CTG GAC ACC TCT GCT TC	NM_011452.2
Tap1	CTG CTC TCC CTC TAC CCC TC<br>CTG AGT GGA GAG CAA GGA GTC	NM_001161730.1
Tap2	GCA GAC GAC TTC ATA GGG GA<br>GTT GCT TCT GTC CCA CAG C	NM_011530.3
Tapbp	ACT GGG AAT GGG ACC TTC TGG<br>ACA CAA CGG GTG CTG GTG TTA G	NM_001025313.1
Tgfb1	GCA ACA ATT CCT GGC GTT ACC TTG<br>AAG CCC TGT ATT CCG TCT CCT TG	NM_011577.1
Ulbp1	GGG GCT TCC TCT TGC TCT GT<br>TTA GAG ACC ATG CCC TGC TTG	NM_029975.2

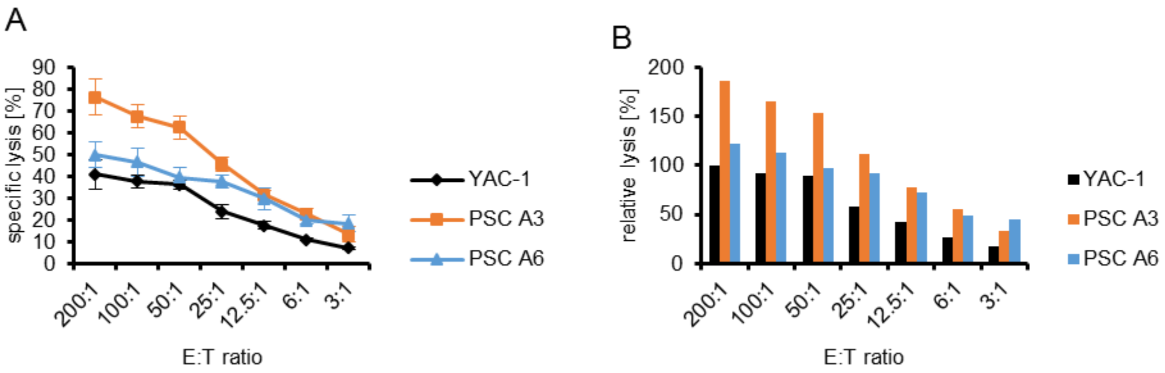

Supplementary Figure 1: Lysis of PSC A3, PSC A6 and YAC-1 target cells by LAK cells of a 129Sv mouse. (**A**) The mean of specific lysis and SEM of YAC-1, PSC A3 and PSC A6 is shown as determined in triplicates in an individual ^{51}Cr-release assay. (**B**) The same data are shown as relative lysis calculated by setting the specific lysis of YAC-1 cells at the highest E:T ratio (200:1) to 100% and adjusting the relative lysis at lower E:T ratios and of other target cells accordingly.

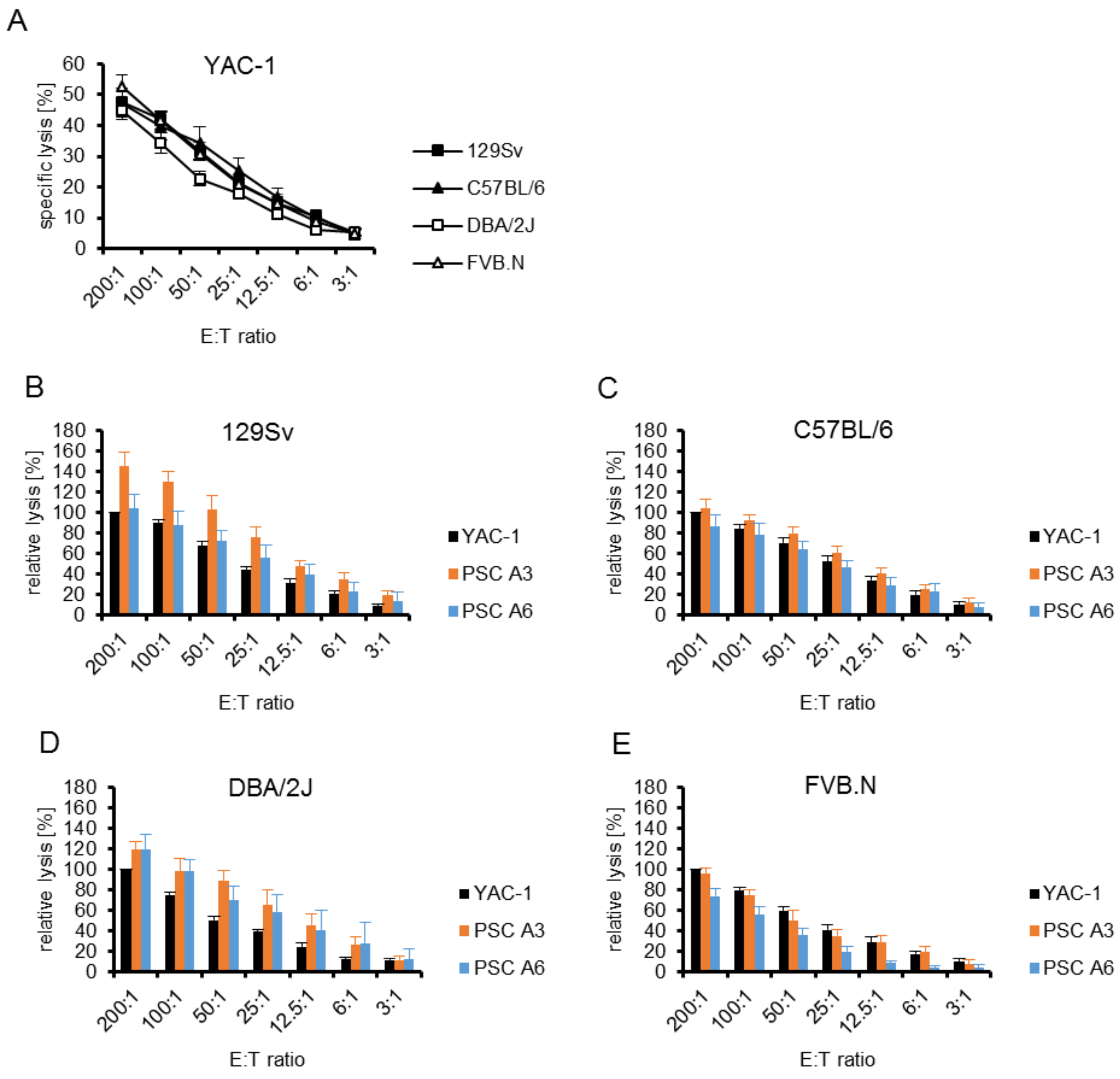

Supplementary Figure 2: Comparison of the killing of PSC A3, PSC A6 and YAC-1 target cells by LAK cells of four mouse strains. (A) A summary of means of specific lysis and SEM of YAC-1 cells by LAK cells from four mouse strains is shown (129Sv n=10; C57BL/6 n=10; DBA/2J n=7: FVB.N n=11). (**B-E**) The same data that are shown in **Figure 1C-F** as specific lysis are displayed here as relative lysis calculated by setting the specific lysis of YAC-1 cells at the highest E:T ratio (200:1) in individual experiments to 100% and adjusting the relative lysis at lower E:T ratios and of other target cells accordingly.

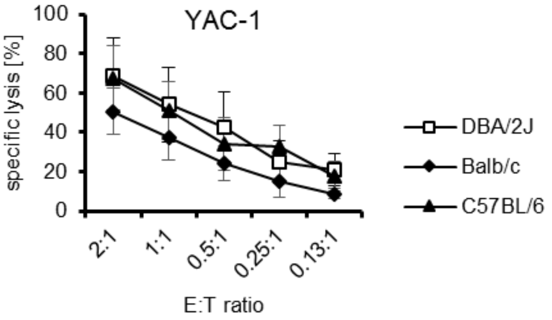

Supplementary Figure 3: Comparison of the killing of YAC-1 target cells by IL-2-activated NK cells of three mouse strains. A summary of means of specific lysis and SEM of YAC-1 cells by NK cells from three mouse stains is shown (DBA/2J, n=3; Balb/c, n=3; C57BL/6, n=3).

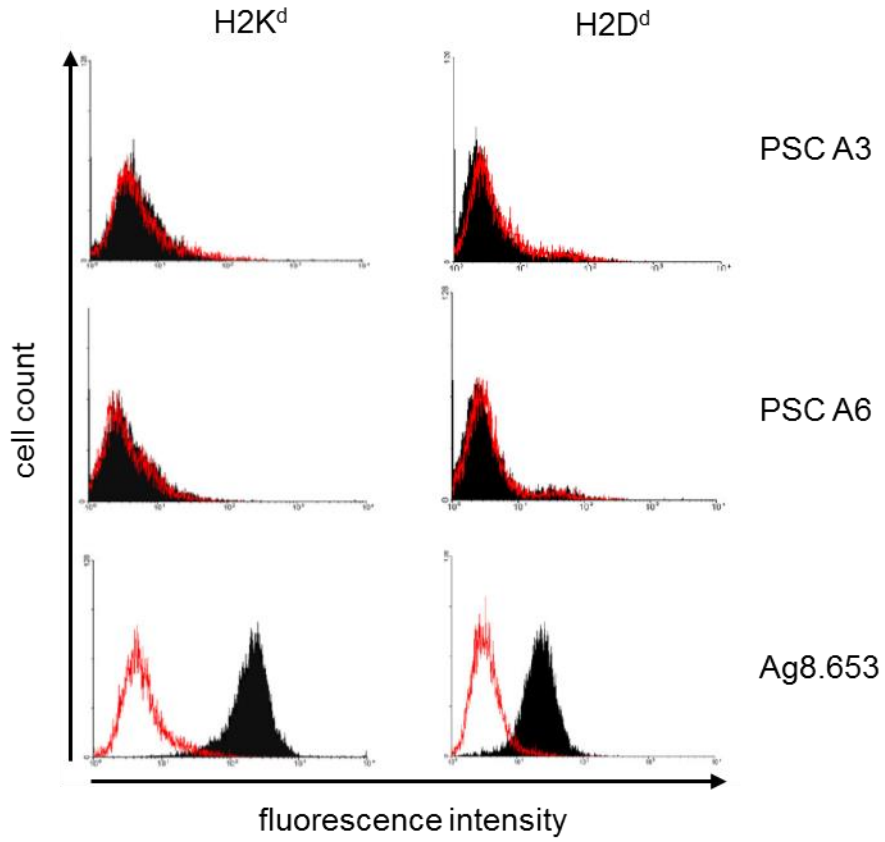

Supplementary Figure 4: Flow cytometric measurement of H2K^d and H2D^d molecules at the plasma membrane of PSC A3, PSC A6 and Ag8.653 cells. An example of a flow cytometric measurement is shown in which the black histograms represent the MHC class I molecules. The red lines indicate the unspecific staining by the isotype control. Ag8.653 cells served as positive control in this experiment.

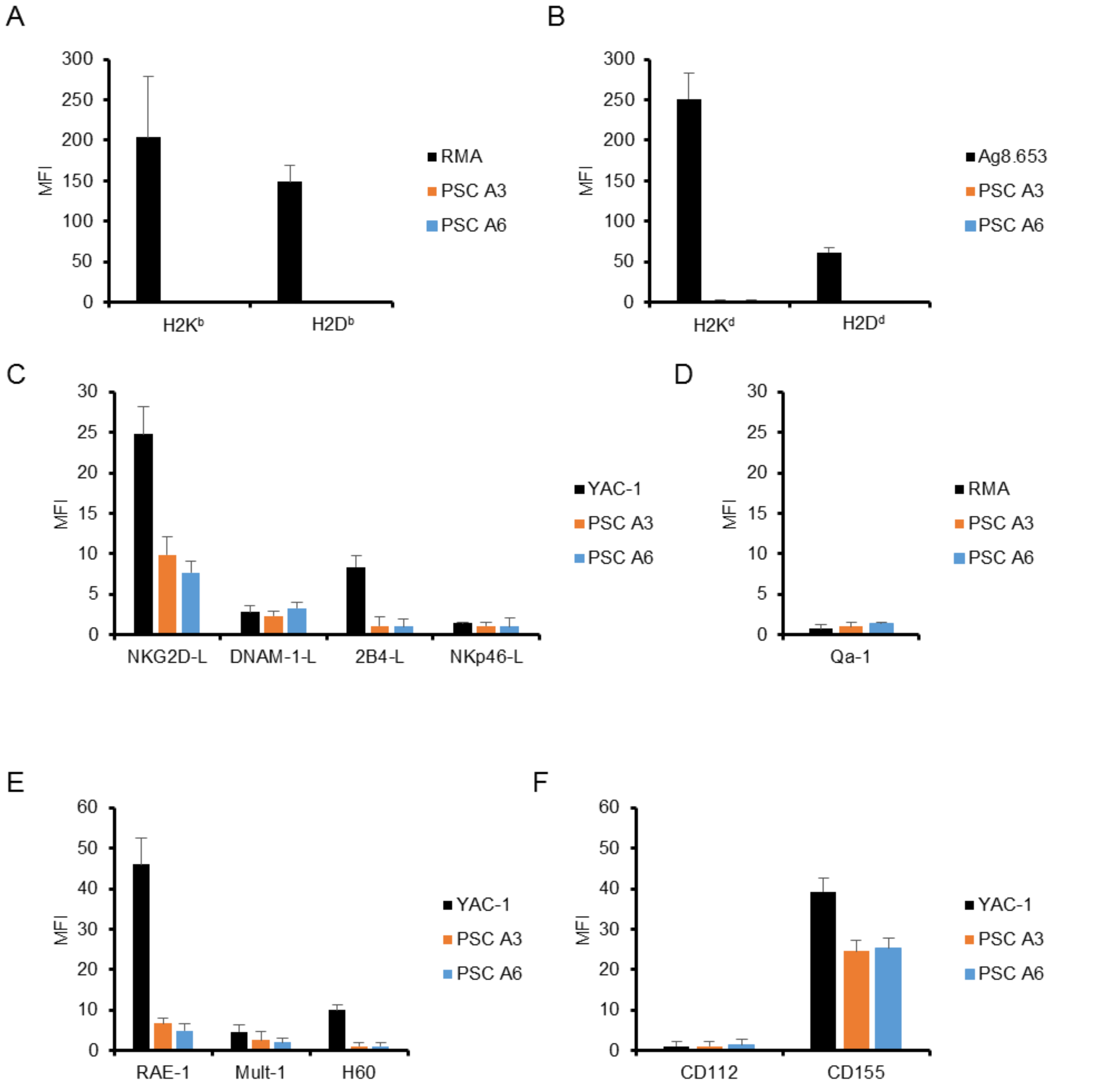

Supplementary Figure 5: Analysis of the MFI of NK receptor ligands on PSC A3, PSC A6 and control cells. The MFI plus SD of the indicated NK receptor ligands is shown as determined by flow cytometry in 3 to 18 individual experiments. The data correspond to **Figure 3** in which the percentage of cells expressing these ligands is shown. (**A**) The MFI of $H2K^b$ and $H2D^b$ molecules has been determined in comparison to RMA cells. (**B**) The MFI of $H2K^d$ and $H2D^d$ molecules has been determined in comparison to Ag8.653 cells. (**C**) The MFI of ligands of the activating NK receptors NKG2D, DNAM-1, 2B4, and NKp46 on the PSCs in comparison to YAC-1 cells has been analyzed by assaying the binding of recombinant receptor-Fc fusion proteins. (**D**) The MFI of the non-classical MHC class I molecule Qa-1 has been tested in comparison to RMA cells. (**E**) The MFI of the NKG2D receptor ligands RAE-1, MULT-1, and H60 has been analyzed in comparison to YAC-1 cells. (**F**) The MFI of DNAM-1 receptor ligands CD112 and CD155 has been tested in comparison to YAC-1 cells.

A

	PSC A3	PSC A6	A3 diff	A6 diff	RMA	spleen DBA/2J	YAC-1
H2D	4,5	3,5	3,2	-2,1	-2,1	-5,4	
H2K	1,6	0,8	3,1	-1,1	-3,7	-5,0	
B2m	0,1	-1,3	2,4	-1,4	-4,7		
Calr	-2,0	-3,0	-2,5	1,8	-3,2		
Canx	-1,3	-2,3	-1,9	1,6	-2,4		
Erp57	-1,2	-2,4	-1,5	1,6	-4,8		
Tap1	2,8	2,4	5,6	0,7	-0,6		
Tap2	4,5	4,7	11,0	8,9	-1,6		
Tapbp	3,7	2,6	5,6	10,9	-1,0		
Psmb9	8,9	8,9	7,8	12,4	1,4		
Psmb8	10,7	10,0	10,3	11,3	0,4		
Qa1b	12,2	11,9	14,0	14,5	-0,8		
Cd80	12,8	10,9	8,7	4,7		4,2	
Cd86	16,6	18,0	10,5	9,5		3,2	
Raet1	3,3	3,6	8,4	9,6			5,4
H60	14,7	12,6	12,1	12,3			6,5
Ulbp1	3,9	5,0	11,2	11,7			5,3
Nectin2	2,1	2,3	7,8	3,8			2,7
Pvr	2,7	3,7	8,7	4,6			6,9
Cd226	8,7	7,4	12,9	9,6		-1,8	11,2
Klrk1	8,5	7,8	13,6	10,6		4,4	13,7

B

	PSC A3	PSC A6	A3 diff	A6 diff	OT-I	placenta	liver	RMA
Serpinb9	8,8	8,5	11,5	14,8	-0,4			
Ido1	4,5	4,8	6,2	9,3		0,4		
Arg1	15,7	13,5	11,8	7,6			-0,4	
Tgfb1	9,0	6,6	10,7	4,7				-1,3

18,0	15,0	12,0	9,0	6,0	3,0	0,0	-3,0	-6,0
low								high

Supplementary Figure 6: Analysis of gene expression patterns in PSC A3 and PSC A6 cells and *in vitro*-differentiated A3 and A6 cells in comparison to control cells or tissues. Heat maps are shown to indicate the expression of genes involved in antigen presentation, T cell and NK cell stimulation (**A**) or the inhibition of cytotoxic cell activity (**B**). The given numbers are means of Δct values (ct of gene of interest – ct of housekeeping gene) of 3 to 4 biological replicates for PSCs, differentiated cells and RMA cells. For the other controls 1 to 3 biological replicates have been analyzed. The color code is shown below and negative values of the Δct (red color) indicate a higher expression of the gene of interest than the housekeeping gene *Hprt*.

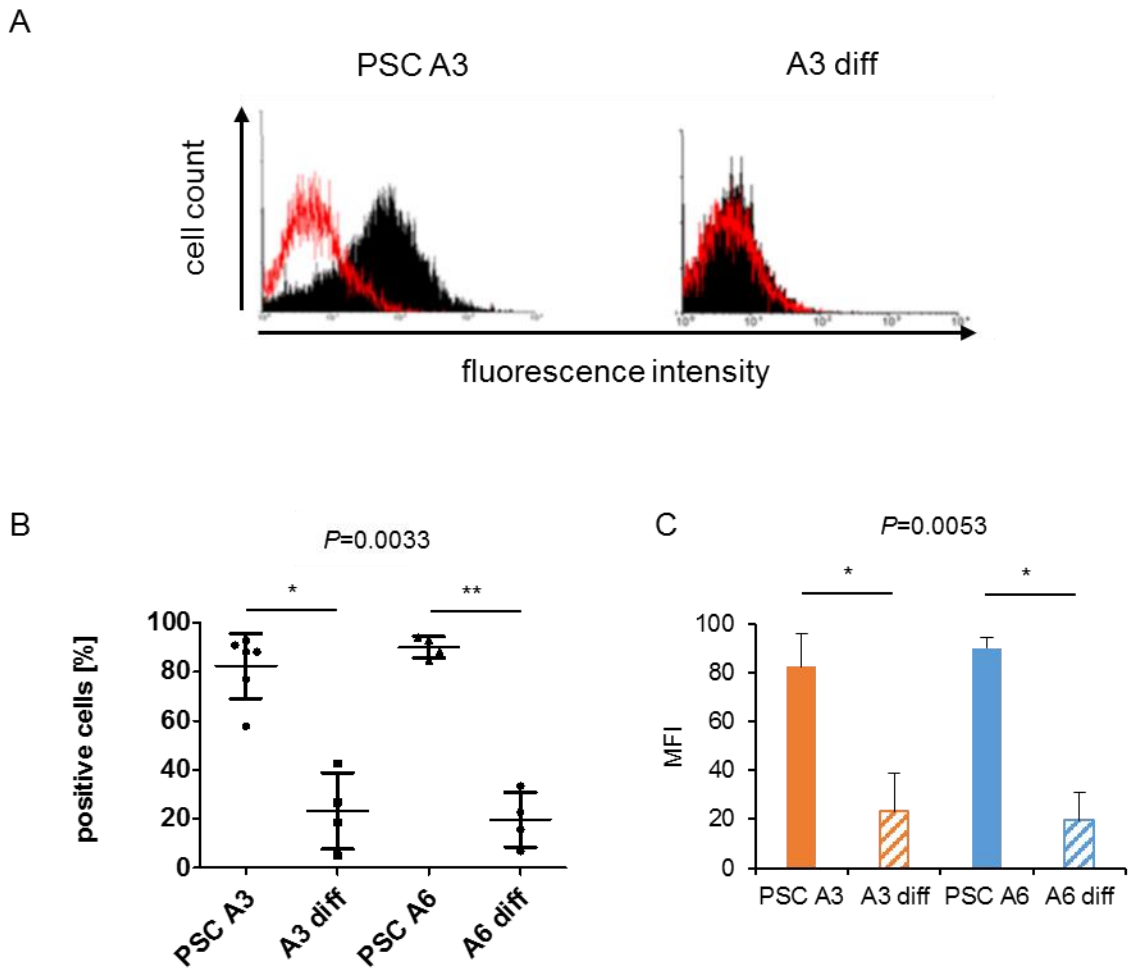

Supplementary Figure 7: Flow cytometric measurement of SSEA-1 molecules on PSCs and *in vitro*-differentiated cells. (**A**) An example of a flow cytometric measurement of PSC A3 and differentiated A3 cells is shown in which the black histograms represent the SSEA-1 expression at the plasma membrane. The red lines indicate the unspecific staining by the isotype control. (**B**) The percentage of cells expressing SSEA-1 is shown as determined by flow cytometry in 4 to 6 individual experiments. In addition, the means ± SD are indicated. (**C**) The MFI plus SD of SSEA-1 is shown. The data correspond to data in panel **B**. The differences between the four cell types in panels **B** and **C** have been analyzed by Kruskal-Wallis tests and the respective P values are indicated. Differences between PSCs and the respective differentiated cell types have been analyzed by Dunn's post hoc test (* P<0.5, **P=<0.01).

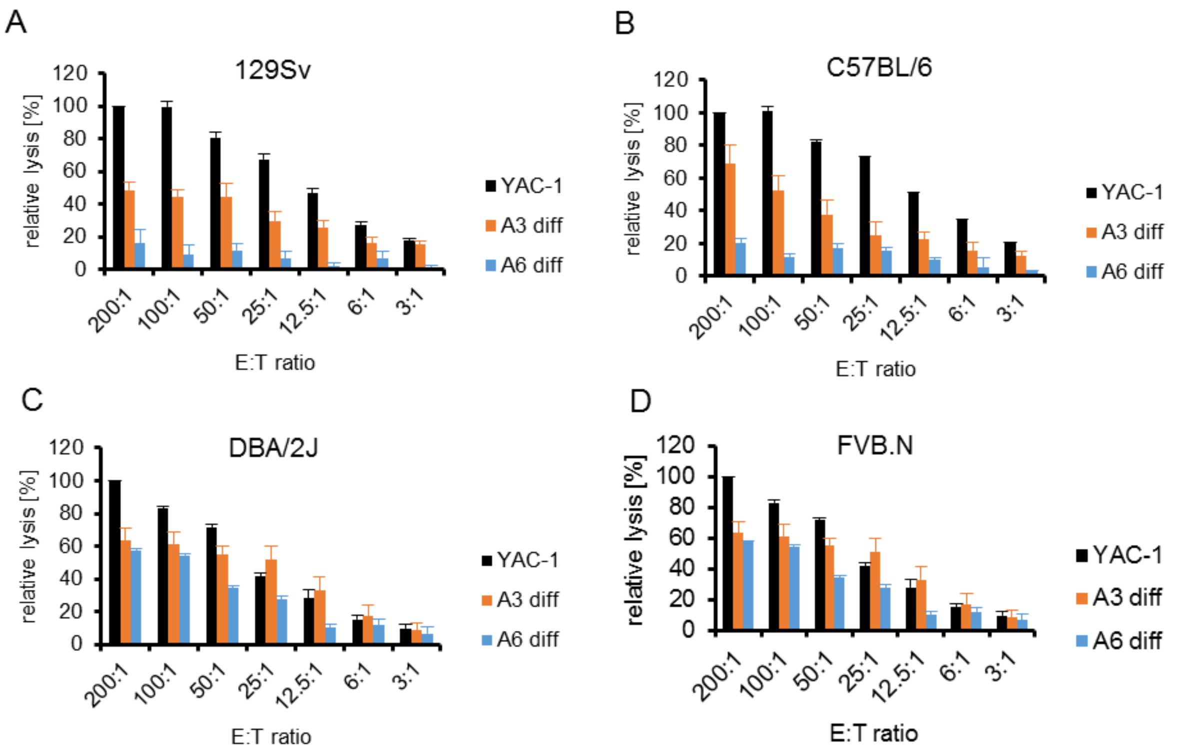

Supplementary Figure 8: Comparison of the relative lysis of differentiated A3, differentiated A6 and YAC-1 control cells by LAK cells of four mouse strains. The same data that are shown in **Figure 4C-F** as specific lysis are displayed here as relative lysis calculated by setting the specific lysis of YAC-1 cells at the highest E:T ratio (200:1) in individual experiments to 100% and adjusting the relative lysis at lower E:T ratios and of other target cells accordingly. The relative lysis and SEM is shown for the three target cell lines YAC-1, PSC A3 and PSC A6 using LAK cells from four mouse strains (129Sv, n=12; C57BL/6, n=5; DBA/2J, n=5: FVB.N, n=6).

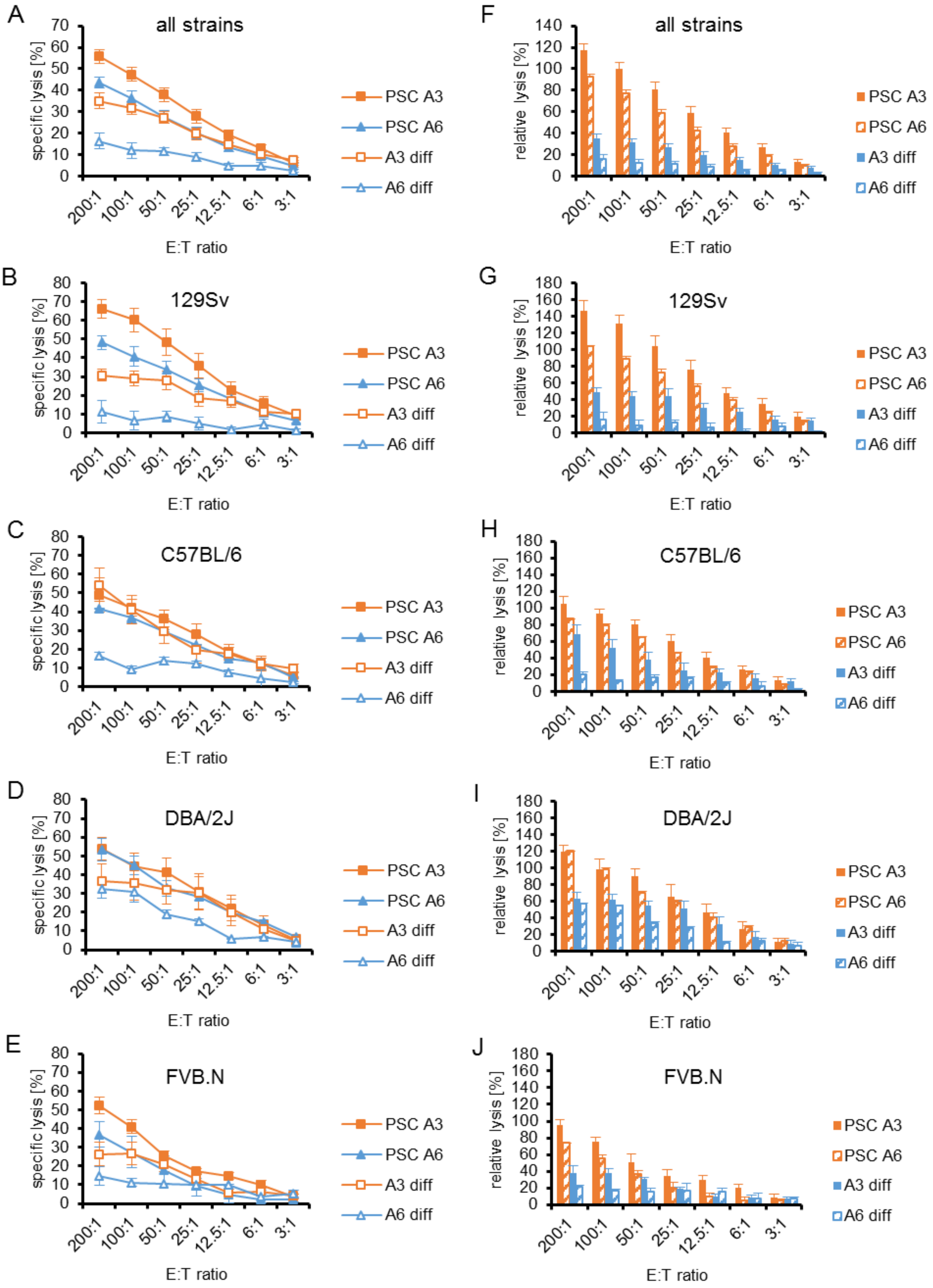
A
all strains
specific lysis [%]
E:T ratio
200:1
100:1
50:1
25:1
12.5:1
6:1
3:1
PSC A3
PSC A6
A3 diff
A6 diff
B
129Sv
C
C57BL/6
D
DBA/2J
E
FVB.N
F
G
H
I
J
relative lysis [%]

Supplementary Figure 9: Comparison of the lysis of PSCs and differentiated A3, differentiated A6 and YAC-1 control cells by LAK cells of four mouse strains. Displayed are the means of specific lysis of PSC A3 and PSC A6 cells in comparison to the differentiated cells derived therefrom (**A-E**) and the relative lysis (**F-J**) calculated by setting the specific lysis of YAC-1 cells at the highest E:T ratio (200:1) in individual experiments to 100% and adjusting the relative lysis at lower E:T ratios and of other target cells accordingly. The results are shown for LAK effector cells from the four mouse strains together (PSC A3, n=18; PSC A6, n=20; A3 diff, n=16; A6 diff, n=12) (**A, F**) and individually, i.e. 129Sv (PSC A3, n=5; PSC A6, n=5; A3 diff, n=6; A6 diff, n=6) (**B, G**), C57BL/6 (PSC A3, n=4; PSC A6, n=6; A3 diff, n=3; A6 diff, n=3) (**C, H**), DBA/2J (PSC A3, n=4; PSC A6, n=3; A3 diff, n=3; A6 diff, n=2) (**D, I**), and FVB.N (PSC A3, n=5; PSC A6, n=6; A3 diff, n=4; A6 diff, n=2) (**E, J**).

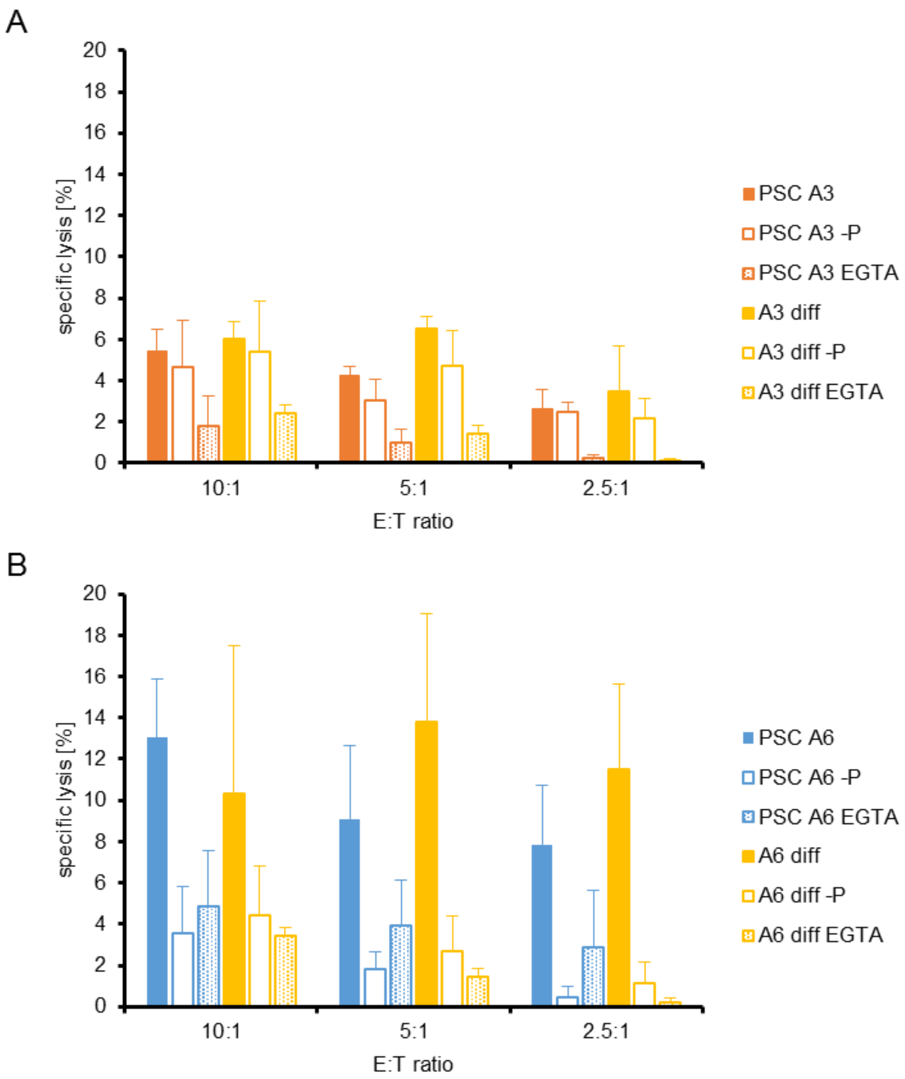

Supplementary Figure 10: Analysis of peptide and calcium dependency of the CTL-mediated killing. A summary of means of specific lysis and SEM of (**A**) A3 and (**B**) A6 cells by CTLs from OT-I mice is shown (n=3 to 6) as measured in ^{51}Cr release assays. During the 4-hr cytotoxicity test the SIINFEKL peptide (0.5 µg/ml) was present or absent (-P). To determine the granule exocytosis dependency of killing, EGTA and $MgCl_2$ were added to some samples.
